# Simulated Heat Waves Affect Cell Fate and Fitness in the Social Amoeba *Dictyostelium discoideum*

**DOI:** 10.1007/s00248-025-02519-y

**Published:** 2025-04-01

**Authors:** Sarena Banu, Katharina C. Wollenberg Valero, Francisco Rivero

**Affiliations:** 1 Centre for Biomedicine, Hull York Medical School, University of Hull, Cottingham Road, Hull, HU6 7RX UK; 2 Energy and Environment Institute, University of Hull, Hull, UK; 3 School of Biology and Environmental Science, University College Dublin, Dublin, Ireland; 4 Conway Institute, University College Dublin, Dublin, Ireland

**Keywords:** Heatwave, Heat stress, *Dictyostelium*, Fitness, Development, Spore yield

## Abstract

The effects of heatwaves at organism and population levels have been widely investigated; however, little is known about how they affect the development of cell populations and the fitness of the resulting organism. Disruptions caused by heatwaves are especially critical during early developmental stages in organisms lacking parental developmental protection or care. Here we use the social amoeba *Dictyostelium discoideum*, a soil microbe with a life cycle that transitions between single-cell and multicellular stages. *D. discoideum* thrives optimally at 22 °C and elevated temperatures impair (27 °C) or completely arrest (30 °C) growth, development, and spore yield. We established a simulated heatwave model in which vegetative cells were exposed to 27 °C for 3 days and studied the effects on the expression of early and cell type specific developmental genes using real-time quantitative PCR. A single heatwave severely impaired the expression of cyclic AMP-dependent early developmental gene markers (*carA*, *acaA*, *pkaR*, *gtaC*, *tgrC1*, and *csaA)* as well as that of prespore markers (*cotB* and *spiA)*, while the expression of the prestalk marker *ecmA* was less affected. When mixed with heat-stressed cells, reporter cells expressing β-galactosidase grown at 22 °C preferentially occupy the spore mass of the fruiting body. Chimera assays of wild-type and reporter cells grown at optimal temperature or subjected to a heatwave confirmed a decreased fitness (contribution to chimeric fruiting bodies). We conclude that exposure of unprotected organisms at the single cell stage to a single heatwave has the potential to negatively impact their ability to cope with environmental extremes.

**Supplementary Information:**

The online version contains supplementary material available at 10.1007/s00248-025-02519-y.

## Introduction

Over the past decade, anthropogenic activities have increased global temperatures by 0.2 °C per decade, with a record surface warming of 1.2 °C in 2022 [1], and this trend is expected to continue. A major consequence of global warming is the rise in the frequency of heatwaves, defined as periods of extremely hot weather lasting three or more consecutive days with daily maximum temperatures 5 °C or higher than the average temperature [1, 2]. Heatwaves significantly impact all living organisms [2, 4]. For example, Smith et al. [5] reported that marine heatwaves caused 44% mortality in gorgonian soft corals in the Western Mediterranean. Similarly, Liang et al. [6] showed that heatwaves impacted forest biodiversity by reducing fish richness by 21% and mobile invertebrate richness by 16%. Population stability for mobile invertebrates and understory algae also decreased by 23% and 17%, respectively. The effects of heatwaves on the microbial flora and fauna remains relatively understudied. Recent marine heatwaves like the Tasman Sea and the equatorial and northeastern Pacific Ocean warm anomalies had a large impact on eukaryotic, archaeal, and bacterial communities niche transitions, particularly affecting phytoplankton communities, whereas dinoflagellates and chlorophytes showed resistance [7, 8]. Heatwave conditions impacted soil microbial communities on grassland soils, preferentially affecting genes related to dormancy, sporulation and metabolism, especially in fungi.[9].

Disruptions caused by heatwaves are especially critical during early developmental stages, when organisms are more vulnerable to environmental changes because of the lack of parental care and buffering conditions [10]. During early development, cells undergo rapid cell division to achieve maturation and protect the embryo. During this period, rapid DNA mutations may occur, impacting development [11]. Heat stress affects embryonic development and alters fitness-related traits such as growth rate and hatching success in birds, reptiles and fish [12, 13]. However, we have limited information on the mechanism by which heatwave conditions affect the ability of cell populations to communicate and differentiate and the consequences for fitness of the organism. In most eukaryotes, rapid cell division and age are major factors that limit the study of heat stress effects at the unicellular level and their impact on multicellular differentiation and fitness of adult individuals [14, 15].

To overcome these limitations, we use the social amoeba *Dictyostelium discoideum* as a model organism with a life cycle that transitions between single cell and multicellular stages. Single-celled (solitary) amoebae feed on bacteria under favorable conditions. During nutrient starvation, cyclic AMP (cAMP) signaling triggers aggregation, resulting in a multicellular mound covered with a cellulose sheath that develops into a migrating slug and subsequently a mature fruiting body. In the slug, cells differentiate into two main cell types: prespore cells (80%) produce spore coat proteins and mature into spores, while prestalk cells (20%) produce extracellular matrix proteins and form a stalk that supports the sorocarp. Spores disperse to survive harsh conditions and, when favorable conditions return, they germinate to restart the life cycle [16, 17]. *D. discoideum* naturally exhibits cooperation and relatedness behaviour, both of which balance the fitness of the organism [18]. Cooperation has been well studied in social amoebae, where stalk cells self-sacrifice to hold the spores aloft, protecting the sorocarp from the harsh environment of the soil and increasing the chance of spore dispersal through invertebrates. Stalk cells thus favour their kin to improve fitness [18–20]. This altruistic behavior is known as inclusive fitness theory or kin selection theory [18–22]. Relatedness is defined as the probability (above random expectation) that an allele is shared by two individuals and in *D. discoideum* it is very high: fruiting bodies in nature are typically clonal due to limited dispersal of spores resulting in patchy distribution of genotypes [18]. Cells are therefore more likely to interact with clonemates, but there is a potential for non-relatives to join an aggregate over millimeter-scale distances. Chimeras formed by mixing of genotypes face benefits and costs, including the risk of cheating, the situation where one genotype contributes more to spore production [18]. There are three cheating strategies: fixed, facultative and obligate. Fixed cheating occurs when one genotype inherently contributes more spores and less stalks. Facultative cheaters force other genotypes to reduce their spore:stalk ratio or increase their own spore:stalk ratio. Obligate cheaters (social parasites) strictly depend on other genotypes to make fruiting bodies but these have not been identified in nature [18, 19].

Although *D. discoideum* is a well-established model for studying development and fitness, there is limited knowledge on the effects of heat stress on those processes. *D. discoideum* lives in forest soil with an optimal growth temperature of 21–22 °C and exhibits a heat shock response at elevated temperatures [23]. Above 27 °C, growth ceases and cells gradually die at 30 °C [23–28]. Heat stress also alters the spore-to-stalk ratio and results in less efficient fruiting body formation [24, 26, 28]. However, it remains unclear how single cells and multicellular structures respond to a heatwave episode, and how these responses influence multicellular populations, development, cooperation with non-stressed populations and overall fitness. To address this knowledge gap, we first investigated the effects of elevated temperatures (27 °C and 30 °C) on growth and development of *D. discoideum*. This information allowed us to establish a simulated heatwave model in which vegetative cells were exposed to 27 °C for 3 days to assess the effects on the expression of developmental marker genes (using real time quantitative PCR, RT-qPCR) and developmental fate and fitness (using reporter strains and chimera assays). We report that a heatwave event had a profound impact on the expression of early developmental as well as cell type specific (particularly prespore) genes, resulting in heat-stressed cell populations being outcompeted by non-stressed cell populations and consequently contributing less spores to chimeric fruiting bodies. Our study suggests that even a single heatwave could negatively impact adaptation to environmental extremes.

## Methods

### *D. discoideum* Strains and Culture Conditions

*D. discoideum* AX2 (referred to as wild-type), an axenically growing derivative of wild strain NC4, was used as control strain. Reporter strains A6-Gal and A15-Gal express β-galactosidase under the control of the constitutive actin6 [29] or actin15 [30] promoters, respectively. Cells were grown on SM agar (Formedium, Swaffham, UK) with *Klebsiella aerogenes* or in AX medium (Formedium, Swaffham, UK), either in Petri dishes or in shaking suspension (100 rpm) at 22 °C [31, 32]. To generate the A15-Gal strain, plasmid pDdGal17-A15-LacZ was retrieved from Dictybase (http://dictybase.org/) and transformed into AX2 as previously described [31, 33]. Transformed strains were selected and grown in medium supplemented with 5 µg/l geneticin (G418; Gibco™, ThermoFisher Scientific, Loughborough, UK).

### *D. discoideum* Growth and Development

For axenic culture growth, cells were inoculated in AX medium and incubated in shaking suspension at the temperatures indicated in each experiment. Growth was monitored daily by counting with a hemocytometer (Marienfeld, Lauda-Königshofen, Germany). For growth on SM agar, 1 × 10^5^ cells were dropped on plates with a *K. aerogenes* lawn and incubated at the temperatures indicated in each experiment. Growth was monitored daily by measuring colony diameter. For development, cells were washed twice in 17 mM Soerensen phosphate buffer, pH 6.0, and either 1 × 10^7^ cells were spread on non-nutrient agar plates or, for synchronous development, 5 × 10^7^ cells were deposited on 47 mm MF-Millipore mixed cellulose ester filters (Merck, Gillingham, UK) as described previously [31, 34] and allowed to develop at the indicated temperatures. Agar plates were documented with a Chemidoc XRS + Imaging System (BioRad, Watford, UK). Development stages were documented with a ZEISS SteREO Discovery.V8 stereomicroscope (ZEISS, Oberkochen, Germany).

### Sporulation Efficiency

To determine sporulation efficiency, cultures were inoculated to achieve a final cell density of 2–3 × 10^6^ cells/ml after growth in suspension for up to 5 days at the indicated temperatures. Cells were washed twice and resuspended in Sorensen buffer and 1 × 10^7^ cells were deposited on 2 × 2 cm squares of non-nutrient agar plates and allowed to develop at 22 °C. After 48 h the agar squares were sliced and spores were harvested in 3 ml of Sorensen buffer. Spores were counted using a hemocytometer and sporulation efficiency was calculated by dividing the number of spores recovered by the initial number of cells plated.

### RNA Extraction and Gene Expression Analysis

RT-qPCR analysis was performed following MIQE guidelines [35]. Vegetative AX2 cells were subjected to a simulated heatwave for 3 days at 27 °C or kept at 22 °C, ensuring cells maintained a density of 1–3 × 10^6^ cells/ml for the entire heat stress period. Cells were washed twice in Soerensen buffer and 1 × 10^8^ cells were allowed to develop synchronously on 47 mm MF Millipore filters (Merck, Gillingham, UK). Cells were scraped from the filters at 4-h intervals for 28 h, washed twice with Soerensen buffer and immediately extracted using the Trizol method (ThermoFisher Scientific, Loughborough, UK). A total of 2 µg of RNA was used for cDNA synthesis with a Bio-Rad iScript cDNA synthesis kit (Bio-Rad Laboratories Ltd., Watford, UK) as per the manufacturer's instructions. RNA quality was verified by spectrophotometric analysis with a NanoDrop (ThermoFisher Scientific, Loughborough, UK) and by agarose gel electrophoresis.

Total cDNA from two experiments, each containing one filter per condition and time point, was used for RT-qPCR analysis using a StepOnePlus™ Real-Time PCR System (Applied Biosystems, Foster City, CA) with iTaq universal SYBR green (Bio-Rad Laboratories Ltd., Watford, UK). Development specific markers, such as early development cAMP-related genes, as well as prestalk and prespore marker genes, were selected from the literature [17]. Corresponding oligonucleotide primers were either obtained from the literature or designed using Primer3 software (https://primer3.ut.ee/), spanning intron–exon junctions where possible, to yield amplicon sizes of 180–200 bp (Table 1). Gene-coding DNA sequences were retrieved from DictyBase (http://dictybase.org/). Primer specificity was verified by BLAST nucleotide search. The amplification efficiency of each primer pair was determined and found within the 90%−110% range for all genes except *gtaC* (Supplemental Fig. 1). Samples were run in two technical replicates and results were analysed using *gpdA* (encoding GAPDH) or *rnlA* (encoding a mitochondrial ribosome RNA component) as reference genes [16]. The results for *gtaC* were corrected to account for the lower amplification efficiency of its primer pair (82%). Ct values of genes of interest were normalized to reference genes and, due to gene expression level differences surpassing a magnitude order of 10, min–max normalized between 0 and 1 based on the minimum and maximum expression values observed at the 22 °C condition.

**Table 1:** *D. discoideum* genes and corresponding primers used for gene expression analysis by RT-qPCR. The genes encoding GPDH (*gpdA*) and a mitochondrial ribosomal RNA component (*rnlA*) were used as reference (housekeeping) genes. The *gpdA* (int) primer pair is intron-specific and was used as a control to exclude the presence of genomic DNA in the cDNA samples

Gene	Forward primer	Reverse primer	Annealing temperature	Reference for primer sequences
*carA*	ATGGTAGTTTTGCATGTTGGTTG	CCAATCCAACACCAATTACCAAC	60^◦^C	[36]
*gtaC*	TATTCTGTGGTACTATGGAAACTC	TGGTAGATGTTGAAGTGGTGGTAG	60^◦^C	This study
*acaA*	CACTTATATGGCGGTTTCAGGTTTAG	CCCCAAACATCAAACTTTGCTCTAC	60^◦^C	This study
*pkaR*	AGAAGAGGAGGAAAGAAACGTTG	TCACCAAAACTACCACCTTCAAATAC	60^◦^C	This study
*csaA*	ACCATCTACAGGTGGTAATGGTC	GGAAATAAATTGTTGGGGATACTG	60^◦^C	This study
*tgrC1*	TCCAACACCAATAGATGCAATATATG	TGAATGGAATAAACAATGTCTCTCTC	60^◦^C	This study
*cotB*	TGAGAATATTAAAATTGGCAGCAC	AATCGTTACTATCTCTTTCAGCAC	60^◦^C	This study
*spiA*	CTGCCGATATTACAGGTCCTCATG	ACCCAATTGTAAACTAATGCAGC	60^◦^C	This study
*ecmA*	AGCATGTGATTGTGATGATGATTG	CACATTTATTGGGGTATGAACAC	65^◦^C	[37]
*gpdA*	GGTTGTCCCAATTGGTATTAATGG	CCGTGGGTTGAATCATATTTGAAC	60^◦^C	[38]
*gpdA (int)*	TGGTTTTGGTCGTATCGGTATG	AACGTGGATTTTGTTACCATTTAC	60^◦^C	This study
*rnlA*	GGTTGTCCCAATTGGTATTAATGG	CCGTGGGTTGAATCATATTTGAAC	60 °C	This study

**Fig. 1:**
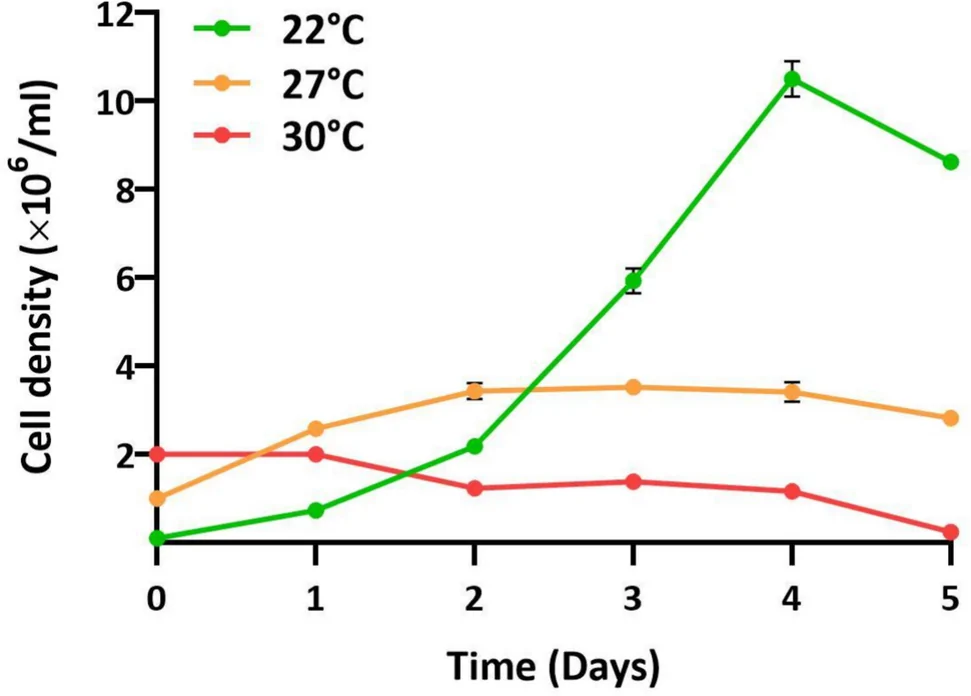
Growth of *D. discoideum* AX2 in suspension in axenic medium at various temperatures. To enable reliable counting, cultures were inoculated at 0.1 × 10^6^ (22 °C), 1 × 10^6^ (27 °C) and 2 × 10^6^ cells/ml (30 °C), grown with shaking and counted daily with a hemocytometer. Data are mean ± SD of 4 (22 °C and 30 °C) or 5 (27 °C) independent experiments

### Cell Fate

To track cell fate, AX2 and A6-Gal or A15-Gal reporter strain cells were cultured clonally at 22 °C or 27 °C for 3 days. Cells were washed twice and resuspended in Soerensen buffer, then 2% of reporter cells were mixed with AX2 cells and 1 × 10^8^ cells were allowed to develop synchronously on 47 mm MF Millipore filters (Merck, Gillingham, UK). Filters with development structures were fixed by first soaking for 5 min then by completely submerging for 20 min in 2% glutaraldehyde. Washing and staining with X-gal was performed as described by Dingermann et al. [39]. Images were taken using a ZEISS SteREO Discovery.V8 (ZEISS, Oberkochen, Germany).

### Fitness Assay

To determine fitness, AX2 and A6-Gal or A15-Gal reporter strain cells were grown in suspension at 22 °C or 27 °C for 3 days. Cells were washed twice and resuspended in Soerensen buffer at a final density of 1 × 10^8^ cells/ml. Cells of different conditions were mixed in a 1:1 ratio and 1 × 10^7^ cells were deposited on 2 × 2 cm squares of non-nutrient agar plates and allowed to develop at 22 °C. After 48 h, the agar squares were excised and spores were harvested and counted as described earlier. Reporter strain spores were visualized after fixation in 4% formaldehyde and staining with X-gal in suspension [40, 41].

Fitness (number of spores produced per deposited cell) was calculated as described by Buttery et al. [32]. Following parameters were collected: n_s_ = total number of spores recovered; n_c_ = total number of cells deposited on agar (1 × 10^7^); p_s_ = fraction of labeled spores; and p_c_ = initial fraction of labeled cells (in our experiments p_c_ = 0.5 because the strains were mixed in equal proportions). p_s_ was corrected for the fraction of unlabelled spores (11% on average) determined in clonally developed A6-Gal cells. Group fitness was then calculated for each experimental condition (temperature and either mix or clonal population) as W = n_s_/n_c_. Strain fitness refers to the fitness of a strain (parental or reporter) within a mix and was calculated as w_A_ = (n_s_p_s_/n_c_p_c_) for the reporter strain and w_B_ = [n_s_(1-p_s_)] / [n_c_(1-p_c_)] for the parental strain. w_A_ is therefore the number reporter spores per deposited reporter cell and, similarly, w_B_ is the number parental spores per deposited parental cell.Relative within-group fitness of the reporter strain is calculated as v = p_s_/p_c_ and is dimensionless. A v > 1 would indicate that the reporter strain is more abundant among spores than in the initial cell population and vice versa if v < 1.

### Statistical Analysis

Statistical analysis was performed using GraphPad Prism v8.0.1 (La Jolla, CA, USA) or R (Version 4.2.0) packages [42]. Data were collected from at least three independent experiments, except for the gene expression analysis (two replicates). For colony size and fruiting body number data, two response variables were analyzed using nonparametric generalized linear mixed models (GLMMs) after determining that residuals were not normally distributed. RT-qPCR data was analyzed using a beta regression approach to accommodate the bounded nature of the data due to min–max normalisation; a GLMM was fitted using the glmmTMB package in R [43]. For other experiments, data distributions were assessed using the Shapiro–Wilk normality test and Q-Q plots and based on the results of the normality tests, parametric or non-parametric tests were applied to analyze differences. The significance level (α) for all tests was set at 5%. The specific statistical tests used are mentioned in the figure legends.

## Results

### Heat Stress Impairs Growth and Development of D. discoideum

As a first step to establish a heatwave model to be applied to *D. discoideum*, we determined the effect of above-optimum temperatures on growth and development. Two above-optimum temperatures, 27 °C and 30 °C, were chosen based on previous studies [25, 44] and were applied to axenic *D. discoideum* cultures in suspension for 5 days (Fig. 1). At the optimal temperature (22 °C), cells grew with a doubling time of 11 h during the exponential phase and reached densities of 10.5 × 10^6^ cells/ml after 4 days. At 27 °C growth was severely reduced (17 h doubling time) and cultures reached densities of only 3.5 × 10^6^ cells/ml. No growth was observed at 30 °C, and in fact cell densities began to decrease after 2 days (Fig. 1). We tested recovery after 3 days of heat stress by transferring the cells to a Petri dish and incubating at 22 °C. Cells cultured at 30 °C did not recover, whereas cells maintained at 27 °C populated the plate in a few days.

**Fig. 2:**
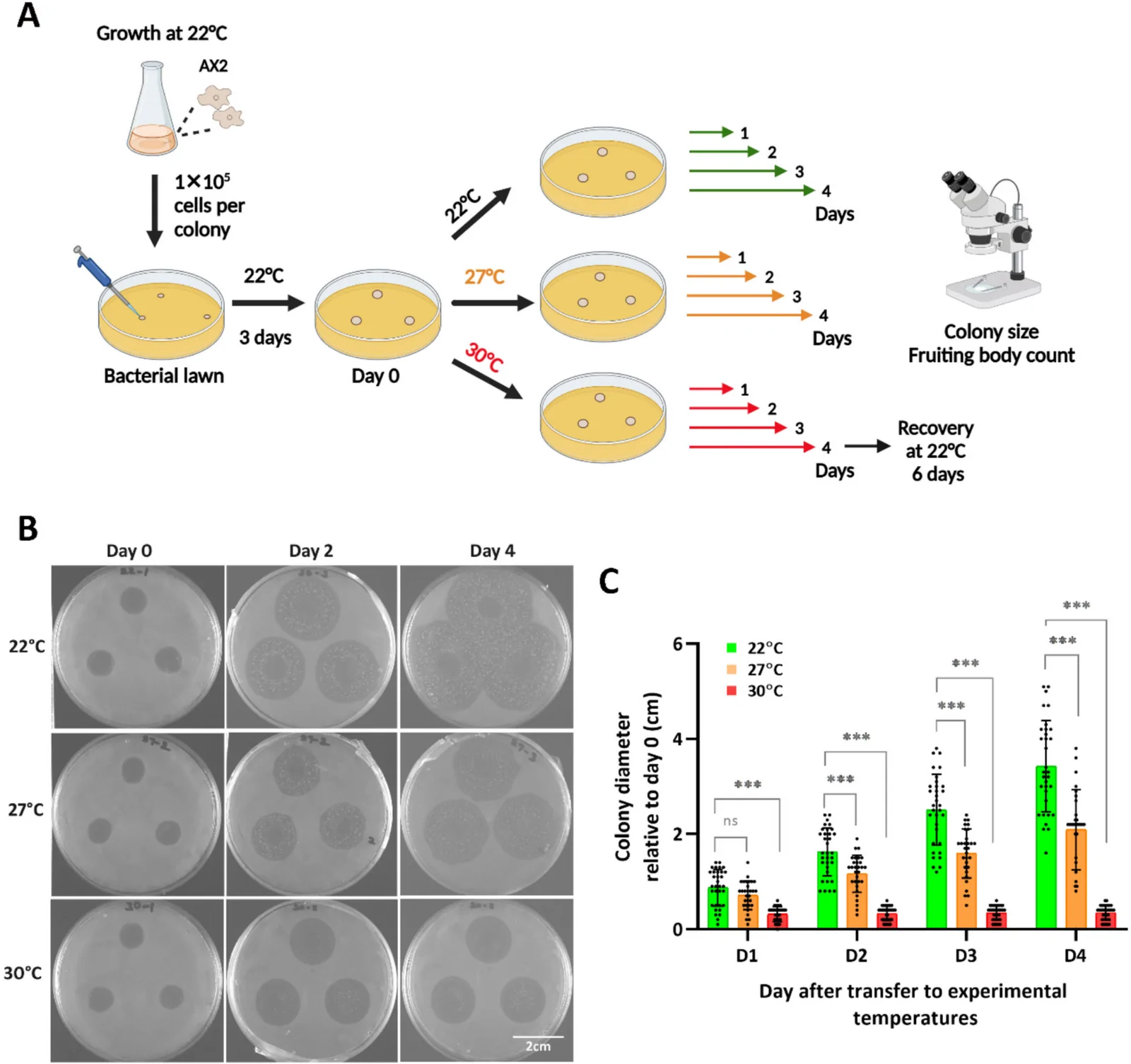
Effect of temperature on *D. discoideum* AX2 colony growth on agar with a *K. aerogenes* lawn. A. Each colony (3 colonies per plate) was inoculated with 1 × 10^5^ cells and allowed to grow at 22 °C for 3 days to become visible before being transferred to the corresponding temperature (Day 0) for 4 days. Created in BioRender. Wollenberg Valero, K. (2025) https://BioRender.com/fp1zdm2. B. Morphology and size of colonies over the course of the experiment. Images of representative agar plates at selected time points. C. Colony growth rate. Colony diameters were measured daily and the diameter at Day 0 was subtracted. Dots represent individual colony measurements. Data are presented as the mean ± SD of individual colonies on two replicate plates from three (30°) to five (22 °C and 27 °C) independent experiments. ***P < 0.001; ns, not significant. Data was analyzed using a non-parametric GLMM followed by an post hoc test. Only comparisons within days are shown; see Supplemental Table 1 for the complete statistical analysis

We next investigated the effects of above-optimum temperatures on *D. discoideum* colonies growing on agar plates in the presence of bacteria, where amoebae can feed and complete the development cycle as they exhaust the bacteria. Colonies were formed by dropping 1 × 10^5^ cells on SM agar plates with a *K. aerogenes* lawn and allowed to grow at 22 °C until visible (approximately 12 mm after 3 days), upon which the plates were transferred to 27 °C or 30 °C or maintained at 22 °C for 4 more days (Fig. 2A). As expected, at 22 °C colonies grew steadily at a rate of 8.5 mm/day, reaching a diameter of 34 ± 9.62 mm at day 4 (Fig. 2B, C). Colony growth was significantly reduced to 4.9 mm/day at 27 °C, reaching a diameter of 20 ± 8.5 mm at day 4 (*P* < 0.001 relative to 22 °C). Colony growth was completely arrested at 30 °C (*P* < 0.001, day 1 through 4).

**Fig. 3:**
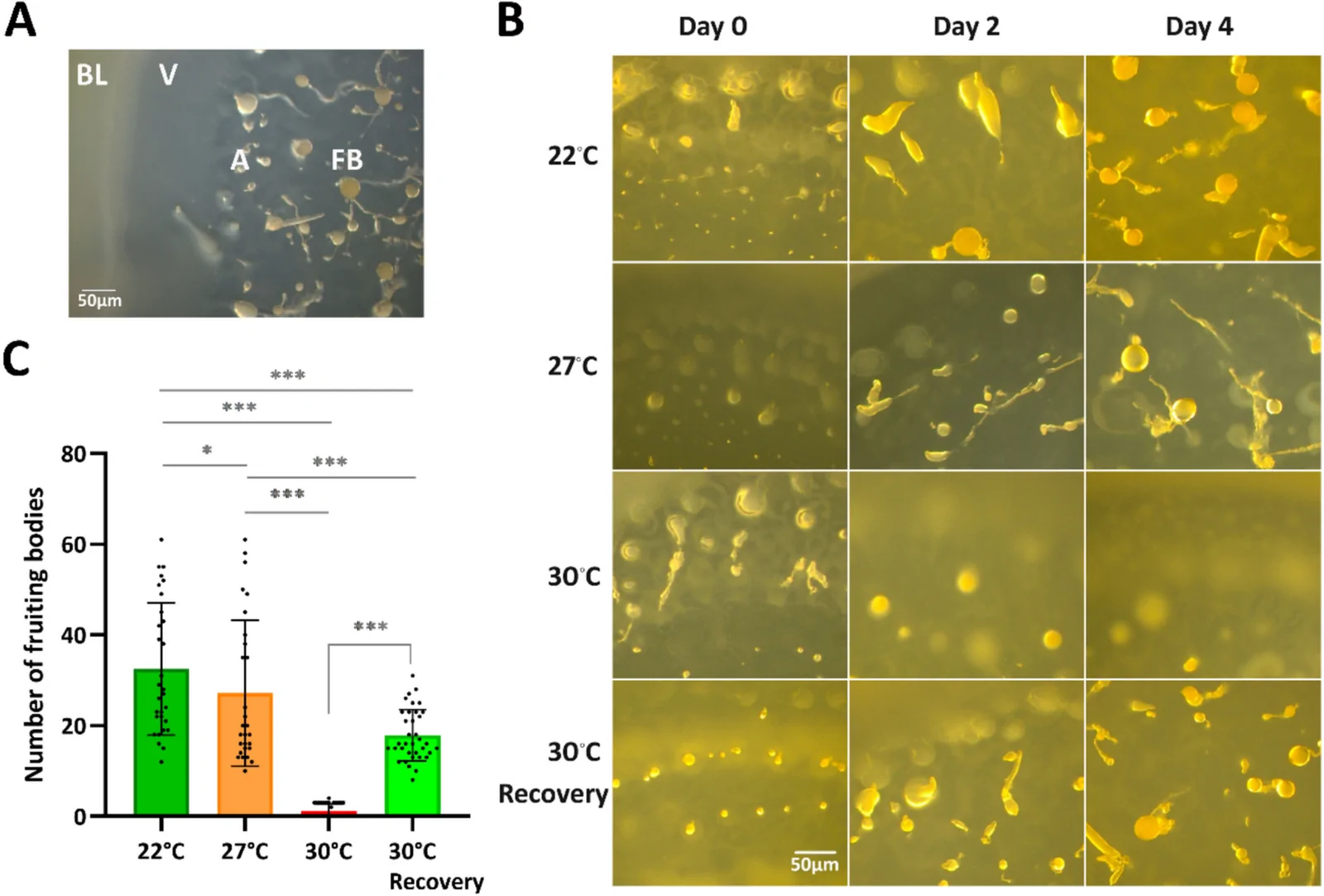
Development of *D. discoideum* AX2 on agar with a *K. aerogenes* lawn at various temperatures. Colonies were formed as in Fig. 2A. For recovery, the plates grown at 30 °C for 4 days were transferred back to 22 °C for 6 days. A. Typical feeding front of a colony on a bacterial lawn. All stages of the *D. discoideum* development are recapitulated in this region. BL, bacterial lawn; V, vegetative cell zone; A, aggregation zone; FB, fruiting bodies. B. Morphology of fruiting bodies. Images were taken with a ZEISS stereomicroscope at the indicated times after transfer to the corresponding temperature. C. Number of fruiting bodies. Fruiting bodies were counted from a 100 mm^2^ area near the feeding front of the colony at day 4. Data are presented as mean ± SD of 28 to 36 colonies from three (30 °C) to five (22 °C and 27 °C) independent experiments. ***P < 0.001; ns, not significant. Data was analyzed using a non-parametric GLMM followed by a post hoc test. See Supplemental Table 2 for the complete statistical analysis

We examined the morphology of the fruiting bodies produced near the feeding front of colonies growing on agar plates with a bacterial lawn at different temperatures. The feeding front is the zone of the colony where amoeba feed on bacteria and divide, and immediately behind starve and begin to aggregate and develop, allowing observation of all stages of the life cycle (Fig. 3A). Development was comparable at 22 °C and 27 °C, with multicellular structures of typical size and morphology, whereas at 30 °C there was very little progress into multicellular structures (Fig. 3B, compare to Fig. 3A). At 22 °C the colonies produced 33 ± 15 fruiting bodies in a 100 mm^2^ area of the feeding front. Heat stress at 27 °C had a modest impact on the number of fruiting bodies (27 ± 16; *P* = 0.0198), whereas heat stress at 30 °C resulted in a dramatic reduction in fruiting body formation (1 ± 2; *P* < 0.0001) (Fig. 3C). To verify whether the colonies grown at 30 °C were still viable after 4 days, they were transferred back to 22 °C for 6 days. Under those conditions the colonies resumed development, with multicellular structures similar to those of colonies grown at 22 °C and 27 °C and produced 18 ± 6 fruiting bodies (P < 0.0001 relative to 30 °C). Based on those results, we chose 27 °C as the heat stress temperature for further experiments.

### Effects of Heat Stress Duration on D. discoideum Development and Spore Yield

**Fig. 4:**
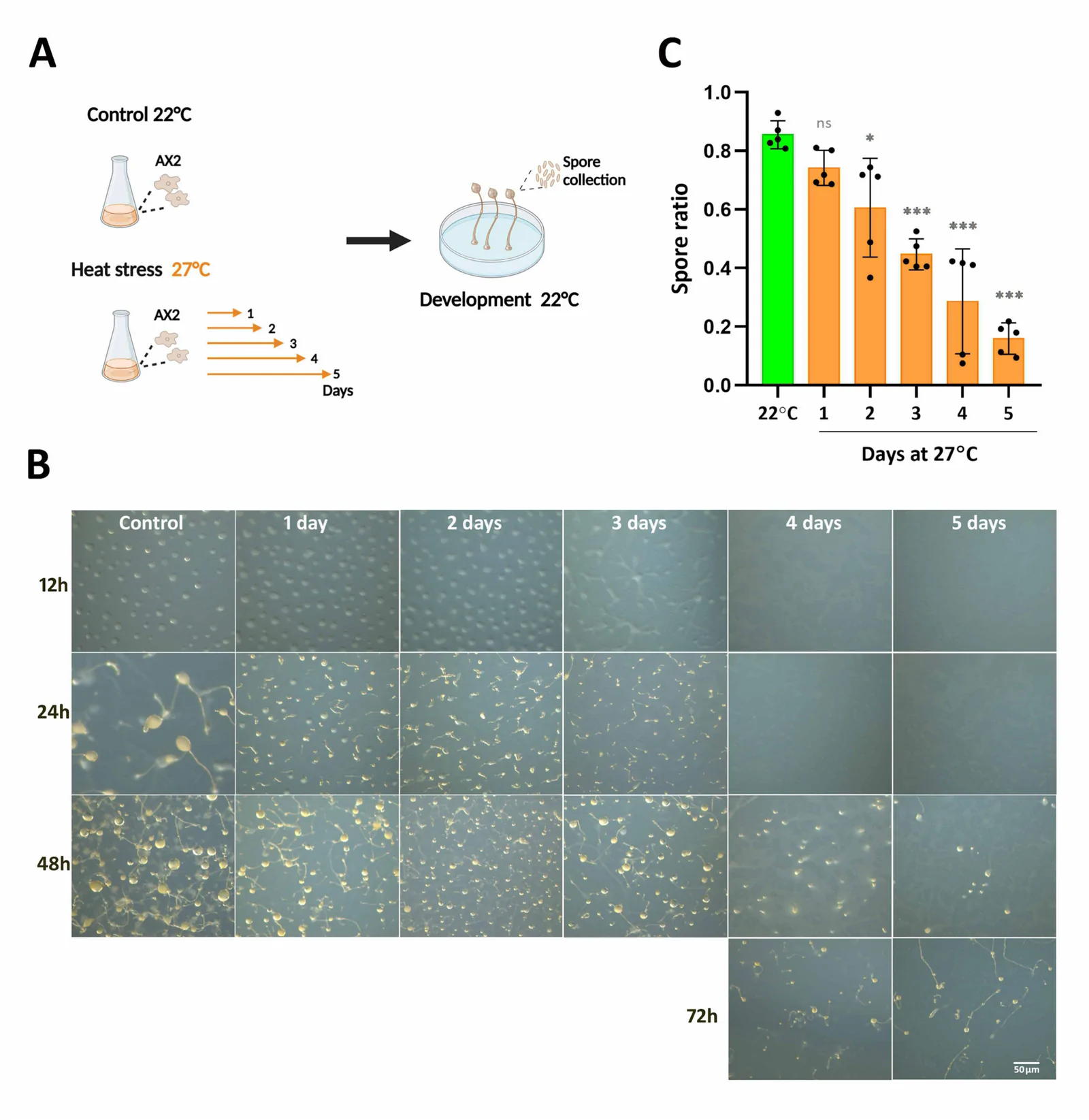
Effect of heat stress duration on development and spore yield. A. Vegetative cells were cultivated in suspension at 27 °C and samples were collected daily for up to 5 days, then allowed to develop on non-nutrient agar plates at 22 °C. Control cells were grown and developed at 22 °C. Created in BioRender. Wollenberg Valero, K. (2025) https://BioRender.com/vl4yh1g. B. Development on non-nutrient agar of control cells and cells grown at 27 °C for the indicated days. Images were taken with a ZEISS stereomicroscope at the indicated time intervals. C. Spore yield. Spores were collected and counted from agar plates at the end of development. Spore ratio is calculated as the number of spores divided by the number of cells plated. Data are mean ± SD of 5 independent experiments, each run in triplicate (three plates per condition). Each dot represents the average of the three plates. ***P < 0.001; *P < 0.05; ns, not significant, relative to 22 °C. ANOVA followed by Tukey’s test. See Supplemental Table 3 for the complete statistical analysis

Having established the above-optimum temperature of 27 °C, we sought to determine the effects of heat stress periods of increasing duration on the developmental process. Vegetative cells were cultivated in suspension at 27 °C and samples were collected every 24 h for up to 5 days, then allowed to develop on non-nutrient agar at 22 °C (Fig. 4A). We chose non-nutrient agar conditions in order to dissociate starvation and development from the concomitant growth that takes place on nutrient agar with bacteria. We observed that after heat stress periods of up to 3 days, cells developed with a timing similar to cells grown at 22 °C, except for a modest delay after 3 days of heat stress. However, heat stress of up to 3 days resulted in smaller fruiting bodies. Vegetative cells subjected to heat stress for more than 3 days showed a considerable development delay and only produced few small fruiting bodies (Fig. 4B and Supplemental Fig. 2). Cells subjected to heat stress for more than 3 days did not show any signs of development at 27 °C on non-nutrient agar for up to 30 h (Supplemental Fig. 3).

The effects of heat stress on fruiting body morphology prompted us to determine the spore yield (the ratio of spores to the vegetative cells allowed to develop) after each time period at 27 °C. At 22 °C the spore ratio was 0.86 ± 0.05, and it decreased progressively as the length of the heat stress period increased, down to 0.16 ± 0.05 (*P* < 0.001) after 5 days (Fig. 4C).

### Heatwaves Cause a Reduction in the Expression of Developmental Genes

**Fig. 5:**
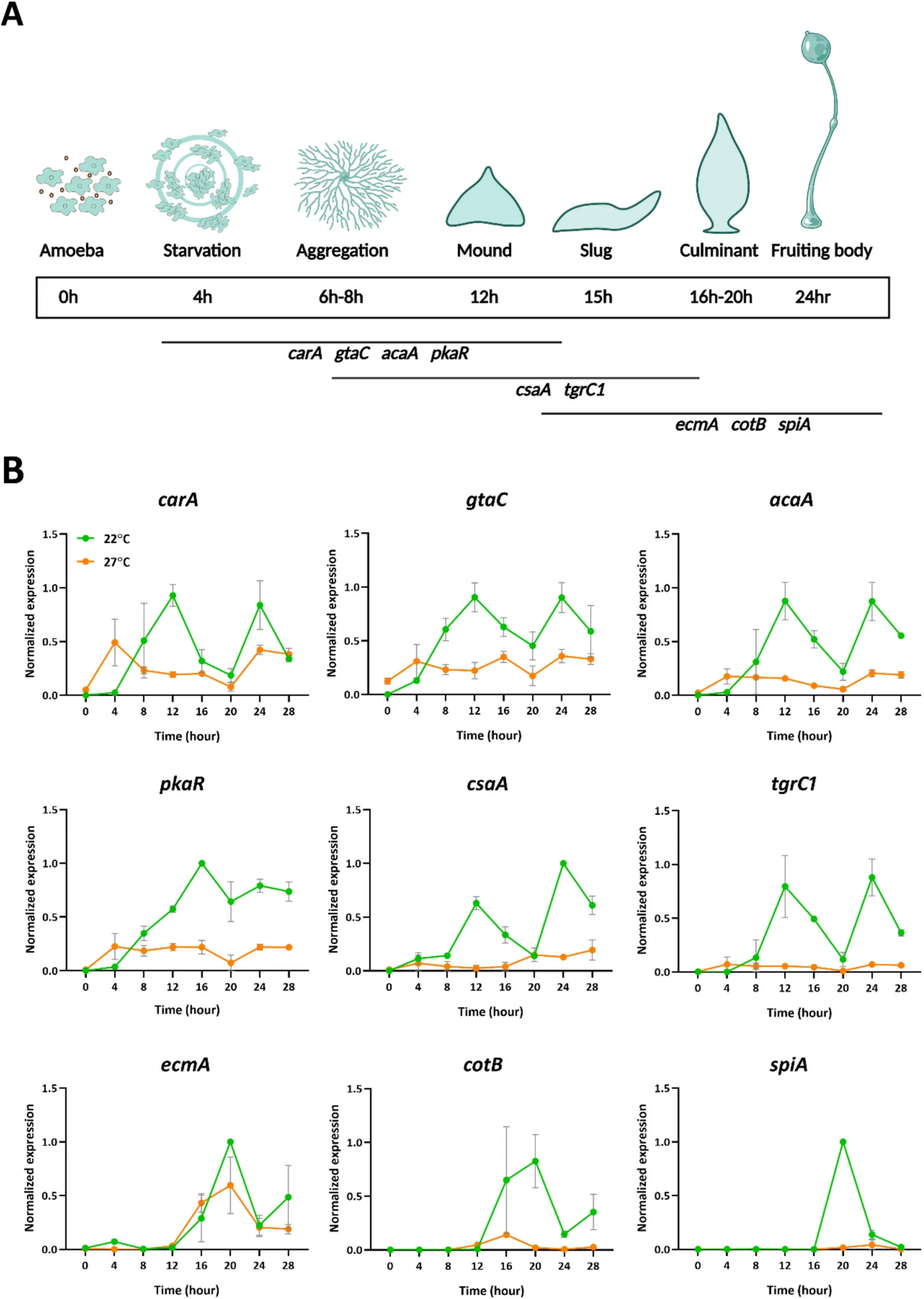
Effects of a heatwave on expression of developmental marker genes. Vegetative AX2 cells were grown at 22 °C or subjected to a simulated heatwave for 3 days at 27 °C and allowed to develop on nitrocellulose filters at 22 °C. Cells were scraped from the filters at the indicated times and used for RNA extraction and RT-qPCR. A. Expression patterns of the developmentally regulated genes analyzed in this study. Created in BioRender. Wollenberg Valero, K. (2025) https://BioRender.com/zohhcrl. B. Gene expression levels normalized relative to the *gpdA* reference gene and min–max normalised. Normalisation to the *rnlA* reference gene produced almost identical results. Data are average ± SD of two independent time courses

Based on our results so far, we chose to simulate a heatwave of 27 °C for 3 days on vegetative cells for a detailed study of its effects on the expression pattern of developmentally regulated genes. After the heatwave, cells were allowed to develop synchronously at 22 °C and RNA was extracted at regular intervals for 28 h. The reported patterns of expression of the genes selected for quantitative real-time PCR analysis (Table 1) are schematically depicted in Fig. 5A. The expression of the reference genes *gpdA* and *rnlA* was found uniform across the development cycle and was not significantly affected by the simulated heatwave (Supplemental Fig. 4).

In cells grown and developed at 22 °C early development genes *carA*, *gtaC*, *acaA*, *csaA*, and *tgrC1* showed a similar pattern of expression (Fig. 5B), with a steady increase during the first 12 h, at which point expression decreased to a minimum at the time of culmination (20 h) to increase again later. The pattern of expression of *pkaR* was slightly different, it peaked at 16 h and remained high throughout development. In cells subjected to a heatwave only a very modest increase in the expression of *carA*, *gtaC*, *acaA*, and *pkaR,* and virtually no increase in the expression of *csaA* and *tgrC1* were observed*.* The cell type specific prestalk gene *ecmA* and the prespore specific genes *spiA* and *cotB* were not expressed at early stages but peaked at 20 h under control conditions [36, 45]. In cells subjected to a simulated heatwave this peak was essentially abolished for the prespore genes but, remarkably, was present for the prestalk marker gene, although at relatively lower levels. The results were analyzed using a GLMM (Supplemental Table 4), that revealed a significant effect of temperature on overall gene expression levels (*p* = 0.0497). No significant interaction was found between temperature and developmental stage, suggesting that the effect of temperature was consistent across developmental time points. In conclusion, a heatwave impaired the expression of early developmental genes and of genes characteristic of the prespore lineage but appeared to affect much less the expression of genes of the prestalk lineage.

### Heatwaves Affect Cell Fate During Multicellular Development

**Fig. 6:**
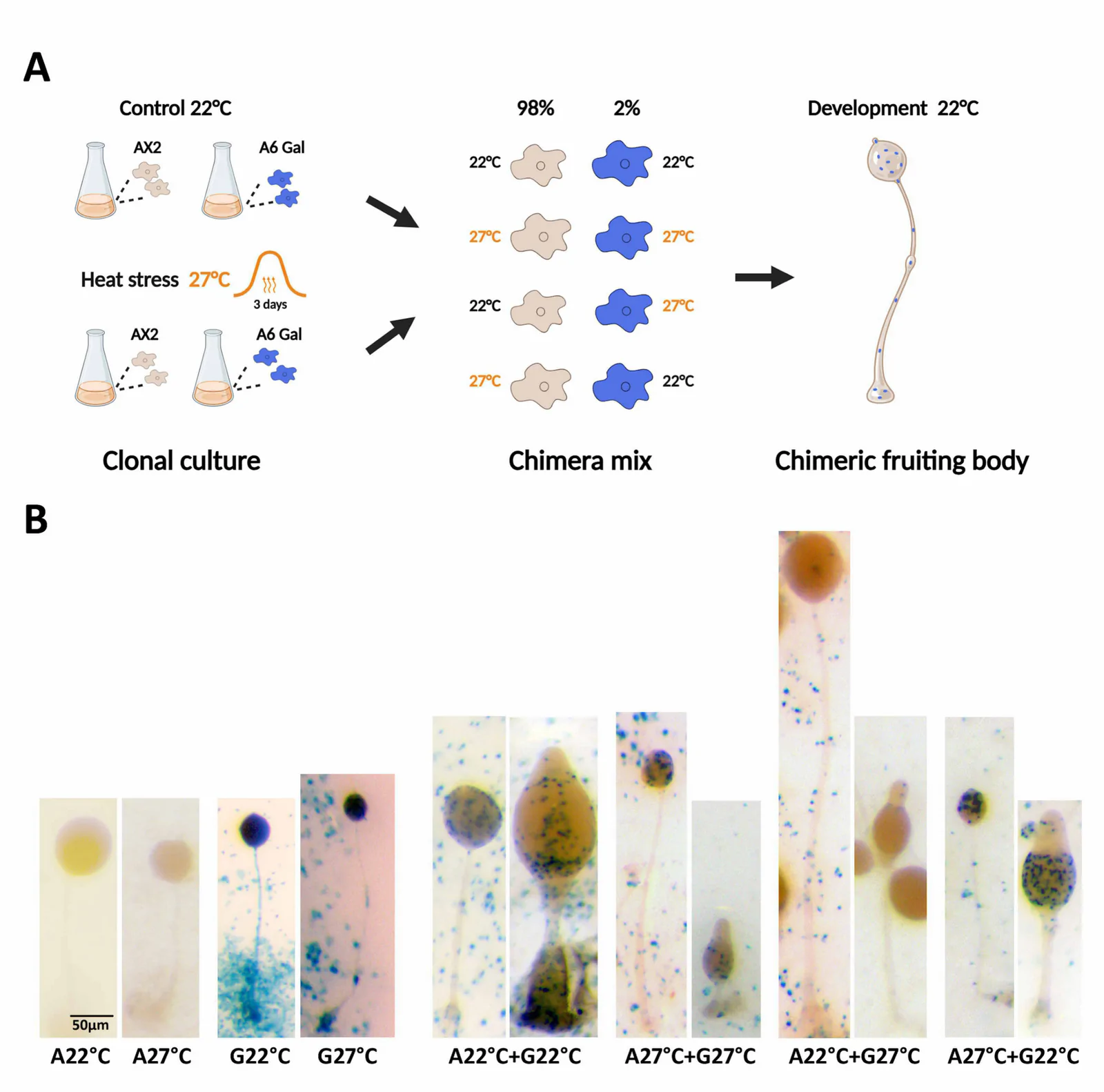
Effects of a heatwave on cell fate during development. A. AX2 and a reporter strain expressing the β-galactosidase gene under the control of the constitutive actin 6 promoter (A6 Gal) were grown at 22 °C or 27 °C for 3 days. Cells were then mixed in various combinations using a 2% proportion of reporter cells, allowed to develop synchronously on nitrocellulose filters at 22 °C and stained. Created in BioRender. Wollenberg Valero, K. (2025) https://BioRender.com/g23fkva. B. Clonal (left) and chimeric (right) culminants and fruiting bodies of AX2 (A) and reporter (G) cells grown at 22 °C or 27 °C for 3 days. Reporter cells appear in blue. Images were taken with a ZEISS stereomicroscope

Heatwaves appear to have different effects on the expression of spore and stalk genes, resulting in small fruiting bodies and a low spore yield. We next investigated whether heatwaves would have an impact on the developmental fate of the cells in a mixed population with cells grown at the optimal temperature. Cell fate was tracked with the AX2-derived reporter strain A6-Gal that expresses the β-galactosidase gene under the control of the constitutive actin 6 promoter. AX2 and the reporter strain were grown at 22 °C or 27 °C for 3 days, mixed in various combinations using 2% of reporter cells, allowed to develop synchronously at 22 °C and stained (Fig. 6A). When both AX2 and A6-Gal were grown at the same temperature, the reporter cells distributed homogeneously throughout the chimeric fruiting body (Fig. 6B, left). However, when the reporter strain cells were subjected to a simulated heatwave and mixed with AX2 cells grown at the optimum temperature, very few reporter cells could be identified in the fruiting bodies. Conversely, when mixed with heat-stressed AX2 cells, reporter cells grown at 22 °C preferentially occupied the spore area of the culminant and the spore mass of the fruiting body (Fig. 6B, right). As expected, cells grown at 27 °C produced small structures compared to those from cells grown at 22 °C. Similar results were obtained with the reporter strain A15-Gal that expresses the β-galactosidase gene under the control of the constitutive actin 15 promoter (Supplemental Fig. 5). These results suggest that cells subjected to a heatwave are at disadvantage when they develop with cells grown at the optimum temperature and, conversely, cells grown at optimum temperature behave as cheaters.

### Effects of Heatwaves on Fitness

Our results suggest that when developing in a mix with cells subjected to a simulated heatwave, cells grown at 22 °C allocate more cells to spores, suggesting that they behave as facultative cheaters. To further investigate this phenotype, we undertook a quantitative fitness analysis [32]. AX2 and A6-Gal cells were grown at 22 °C or 27 °C for 3 days, mixed in various combinations using equal proportions of each strain and allowed to develop at 22 °C (similar to Fig. 6A). Spores were collected, stained and counted.

**Fig. 7:**
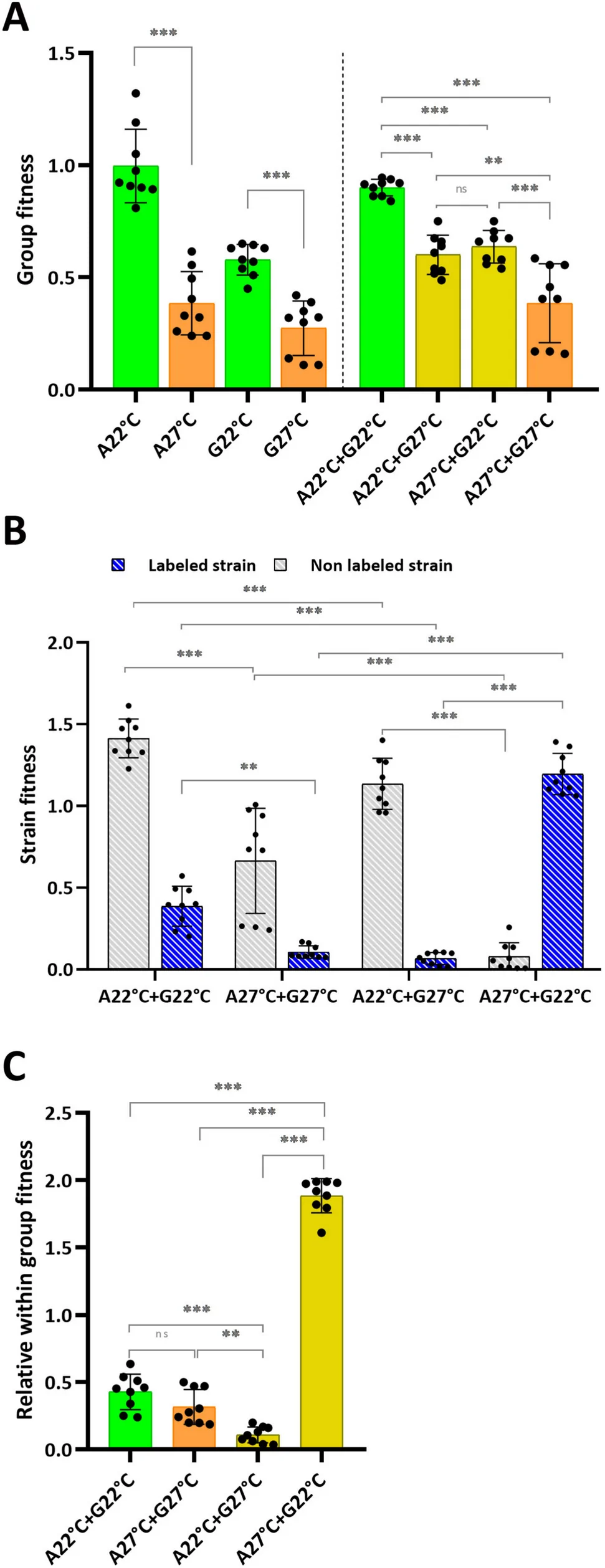
Effects of a heatwave on fitness. Parental AX2 (A) and reporter strain A6-Gal (G) expressing the β-galactosidase gene under the control of the constitutive actin 6 promoter were grown at 22 °C or 27 °C for 3 days. Cells were allowed to develop clonally or mixed 50:50 on non-nutrient agar at 22 °C and spores were collected, stained for β-galactosidase activity and counted. Fitness parameters were calculated as described in the methods section. A. Group fitness of AX2 and A6-Gal in clonal and chimeric mixes. B. Strain fitness in chimera mixes. C. Relative within-group fitness of the A6-Gal strain. All data are presented as the mean ± SD of *n* = 9. ***P < 0.001; **P < 0.01; ns, not significant, relative to 22 °C. ANOVA followed by Tukey’s (A, C) or Sydak’s test (B). See Supplemental Tables 5–7 for the complete statistical analysis

We first analyzed group fitness. When developed clonally, AX2 and A6-Gal strains grown at 22 °C had mean fitness values of 1.00 ± 0.16 and 0.58 ± 0.07 spores per cell, respectively, whereas when grown at 27 °C, spore production significantly reduced to 0.38 ± 0.14 and 0.27 ± 0.12 spores per cell (*P* < 0.001 for both strains), respectively (Fig. 7A). A similar effect was observed in chimera mixes: 0.90 ± 0.04 spores per cell at 22 °C and 0.38 ± 0.18 at 27 °C (*P* < 0.001). In clonal populations and in chimera mixes the scale of the reduction caused by a heatwave was therefore similar. Chimera mixes of cells grown at optimal and heat stress temperatures both produced approximately 0.6 spores per cell, intermediate between the mixes of cells grown at the optimal and the heat-stress temperature. These results indicate that AX2 and A6-Gal both respond similarly to a heatwave and validate the use of the A6-Gal strain as a reporter of AX2.

To examine specific contributions to the temperature effect we undertook a strain-oriented analysis that calculates the fitness of each strain in the mix (Fig. 7B). When developed in equal proportions, AX2 and A6-Gal strains grown at 22 °C had fitness values of 1.41 ± 0.12 and 0.39 ± 0.12 spores per cell, respectively, whereas when grown at 27 °C the fitness significantly dropped to 0.66 ± 0.32 and 0.11 ± 0.04 (*P* < 0.001 for AX2, *P* < 0.01 for A6-Gal), respectively, indicating that, as expected from the clonal populations, fixed cheating has occurred. However, when cells grown at different temperatures were mixed in equal proportions, heat-stressed cells contributed significantly fewer spores (AX2: 0.08 ± 0.09; A6-Gal: 0.07 ± 0.04) than the cells grown at 22 °C (AX2: 1.14 ± 0.16; A6-Gal: 1.20 ± 0.13; *P* < 0.001 for both strains), indicating that facultative cheating of cells grown at 22 °C on cells grown at 27 °C has occurred and is attributable to the heat stress and not to possible genotypic differences between AX2 and A6-Gal. This result was corroborated by the significantly higher relative within-group fitness parameter for the A6-Gal strain grown at 22 °C when developed with AX2 subjected to a heatwave (1.88 ± 0.13; *p* < 0.001 relative to all other conditions), indicating that in those conditions A6-Gal was more abundant among spores than in the initial cell mix (Fig. 7C).

## Discussion

Our study revealed significant impacts of elevated temperatures (27 °C and 30 °C) on growth, development and fitness of *D. discoideum* and identified temperatures above the optimum and the critical maximum thresholds which negatively affect overall fitness. Heat stress reportedly affects the growth of *D. discoideum* in both axenic and bacterial lawn culture conditions at temperatures above 27–28 °C [23–25, 28]. We observed significantly reduced cell and colony growth at 27 °C, which contrasts with an earlier study suggesting no impact on cell growth at this temperature [24]. Consistent with previous studies [23–25], our results show that growth ceases completely at 30 °C, validating 30 °C as the critical maximum temperature for *D. discoideum* growth and development. Interestingly, plaques incubated at 30 °C for 5 days formed fruiting bodies when shifted back to 22 °C, which we attribute to the aggregation and multicellular development initiated before the shift to 30 °C enabling recovery and subsequent fruiting body formation at 22 °C. This observation contradicts earlier findings showing that exposure to 30 °C for more than 10 h causes irreversible morphogenesis arrest at any developmental stage [23]. However, Maeda [46] observed that post-aggregative cells can tolerate shifts to extreme temperatures, such as low temperatures of 5 °C, and still form spores whereas vegetative cells fail to differentiate under similar conditions. These results suggest that *D. discoideum* multicellular stages are more resistant to temperature variation, whereas the unicellular stage is very sensitive to temperature changes. In fact, when unicellular stages were exposed to 27 °C, development at 22 °C was delayed and the spore yield was significantly reduced, indicating that heat stress during the unicellular stage severely impacts *D. discoideum* multicellular development. When *D. discoideum* enters the multicellular life cycle, coordinated cell–cell signaling and aggregation takes place, and the aggregate secretes an extracellular matrix made up of cellulose and proteins. The extracellular matrix provides structural support to the cells as they differentiate, which might help protect the organism from environmental cues [47].

Little is known about the effect of heat stress on signaling pathways regulating development in *D. discoideum*. Our gene expression results show that cAMP dependent genes were highly impaired under heat stress. Nutrient starvation in *D. discoideum* triggers the cAMP signaling pathway through the activation of *acaA* and *carA* genes [17, 48–51]. The receptor encoded by *carA* detects cAMP and stimulates its production via adenylyl cyclase (encoded by *acaA*). Excessive cAMP production activates protein kinase A (PKA), which inhibits *acaA* leading to oscillatory waves of cAMP that attract cells and coordinate aggregation [17, 48–51]. These waves form spiral patterns between 4 and 8 h of development, facilitating cell aggregation. The transcription factor GtaC, upon translocation to the nucleus via cAMP signaling, induces the expression of early developmental genes including *carA*, *acaA*, and *pkaR* and adhesion markers such as *csaA* and *tgrB1*/*tgrC1* [17, 48, 49]. These genes are essential during and after aggregation, contributing to the coordination of cell aggregation and playing crucial roles in cell sorting and differentiation into prespore and prestalk populations [17, 48–52]. Interestingly, *acaA* and *pkaC* null strains show increased survival under heat stress compared to wild-type cells but eventually experience developmental arrest [53]. Mutations in these genes lead to reduced sporulation and abnormal stalk formation [17, 52, 54]. Other stressors, such as UV radiation, oxidative stress and osmotic stress, also affect *D. discoideum* development and have been linked to cAMP signaling and developmental pathways [55, 56]. Under heat stress conditions, cAMP signaling genes peak earlier (at 4 h) compared to the control temperature, suggesting that heat stressed cells sense environmental changes and respond by entering the starvation phase earlier. The downregulation of early developmental genes and aggregation markers after 8 h suggest developmental arrest, which might contribute to the defects observed under 27 °C heat stress conditions.

Previous findings showed that heat stress affects the stalk and spore ratio [25, 26], but its impact on developmental gene expression and signaling has not been investigated. Under heat stress, prestalk and prespore markers are downregulated, which may contribute to the developmental defects and reduced spore ratios we observe. Notably, the expression of the prestalk marker *ecmA* was less affected than that of spore markers, suggesting that heat-stressed vegetative cells favour stalk cell differentiation. This observation implicates a potential kin discrimination mechanism involving a cheating behaviour, where *D. discoideum* favors certain cell types for personal gain under environmental stress and exploits cooperative systems, a mechanism also observed in insects [20]. Cheating can occur during fruiting body formation in both clonal and chimeric conditions observed in laboratory experiments and in natural systems [19, 20, 32, 57]. Cheating behavior may help safeguard organismal fitness and enhance evolutionary success by favouring certain phenotypes [19, 20]. Although defined from a genetics perspective, we found the theoretical frame of cheating very useful to analyze quantitatively the behavior of a population subjected to an environmental situation. Our results showed that wild-type *D. discoideum* AX2 produced more spores than the A6-Gal reporter in both clonal and chimeric mixtures under 22 °C conditions, suggesting a fixed cheating mechanism between the strains [32, 58] and a similar pattern was observed at 27 °C. When heat-stressed vegetative cells were mixed with cells grown at 22 °C, the latter contributed significantly more spores than their initial proportion, while heat-stressed cells contributed minimally to spore formation. This situation where cells grown under optimal conditions benefit by producing more spores while heat-stressed cells are disadvantaged suggests facultative cheating [19, 20, 32]. This division of labour within chimeras highlights how heat-stressed cells, where developmental genes and prespore markers are downregulated, are disadvantaged and contribute less to the spore population. A similar mechanism of selective disadvantage has been observed in mammalian systems, such as cancer biology and embryonic development, where defective cells are selectively removed through autophagy to ensure the survival of healthier cells and maintain overall fitness [59–62]. In other social species, including ants, bees and termites, heat stress and heatwave conditions have been shown to negatively impact colony fitness [63].

Our results show that heatwaves selectively and significantly impact differentiation of the prespore lineage and spore production, a key aspect of the *D. discoideum* reproductive process, suggesting that heat stress could negatively impact evolutionary success. Our results suggest a mechanistic link between reduced fitness and cellular processes at the interface of single-and multicellular developmental stages that involve early onset of the starvation response but overall developmental downregulation with formation of smaller fruiting bodies with potentially less competitive spores. Our results therefore suggest that even a single heatwave below the critical thermal maximum could negatively impact evolutionary trajectories. Wild *Dictyostelium* strains isolated from various terrains other than deserts have evolved multiple times to produce spore-forming fruiting bodies capable of withstanding extreme temperatures, suggesting climate adaptation [64]. Our study demonstrates that simulated heatwaves impact *D. discoideum* by affecting the competitiveness and thus the fitness of the organism, which may propagate negative consequences to the next generation. Further molecular analysis is necessary to better understand the impacts of climate change and potential adaptations in *D. discoideum*, which may apply to other organisms with single-cell stages unprotected against environmental extremes.

## Supplementary Information

Below is the link to the electronic supplementary material.

Supplementary file1 (PDF 538 KB)

## Data Availability

Data is provided within the manuscript or supplementary information files. The datasets generated during and/or analysed during the current study are available from the corresponding author on reasonable request.
