# Supplementary material for: Simulated Heat Waves Affect Cell Fate and Fitness in the Social Amoeba Dictyostelium discoideum
Source: Microb Ecol. 2025 Apr 1;88:21. doi: 10.1007/s00248-025-02519-y (PMC11961495; doi:10.1007/s00248-025-02519-y)
Supplement: Supplementary file 1 [file 248_2025_2519_MOESM1_ESM.pdf]

Supplementary Information

Simulated heat waves affect cell fate and fitness in the social amoeba *Dictyostelium discoideum*

Sarena Banu, Katharina C. Wollenberg Valero, Francisco Rivero*

*Centre for Biomedicine, Hull York Medical School, University of Hull, Hull, United Kingdom, Francisco.rivero@hyms.ac.uk

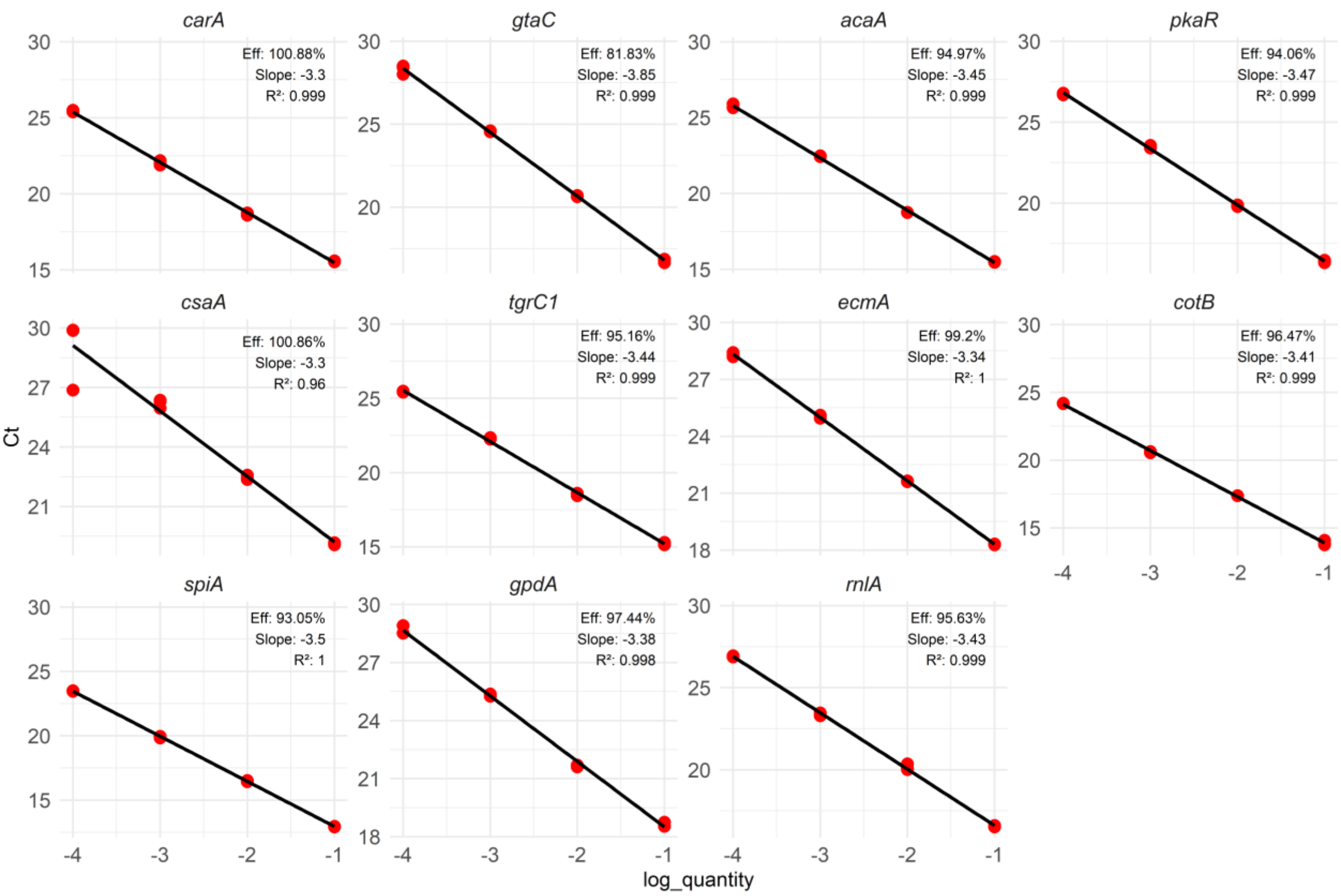

Supplemental Figure 1. Real-time qPCR standard curve analysis for *D. discoideum* developmental and reference genes. cDNA was diluted in a 10-fold series (10^{-1} to 10^{-4}) and the Ct values of three technical replicates per dilution determined. Gene-specific amplification efficiency and goodness of fit for each primer pair were calculated using a linear regression model.

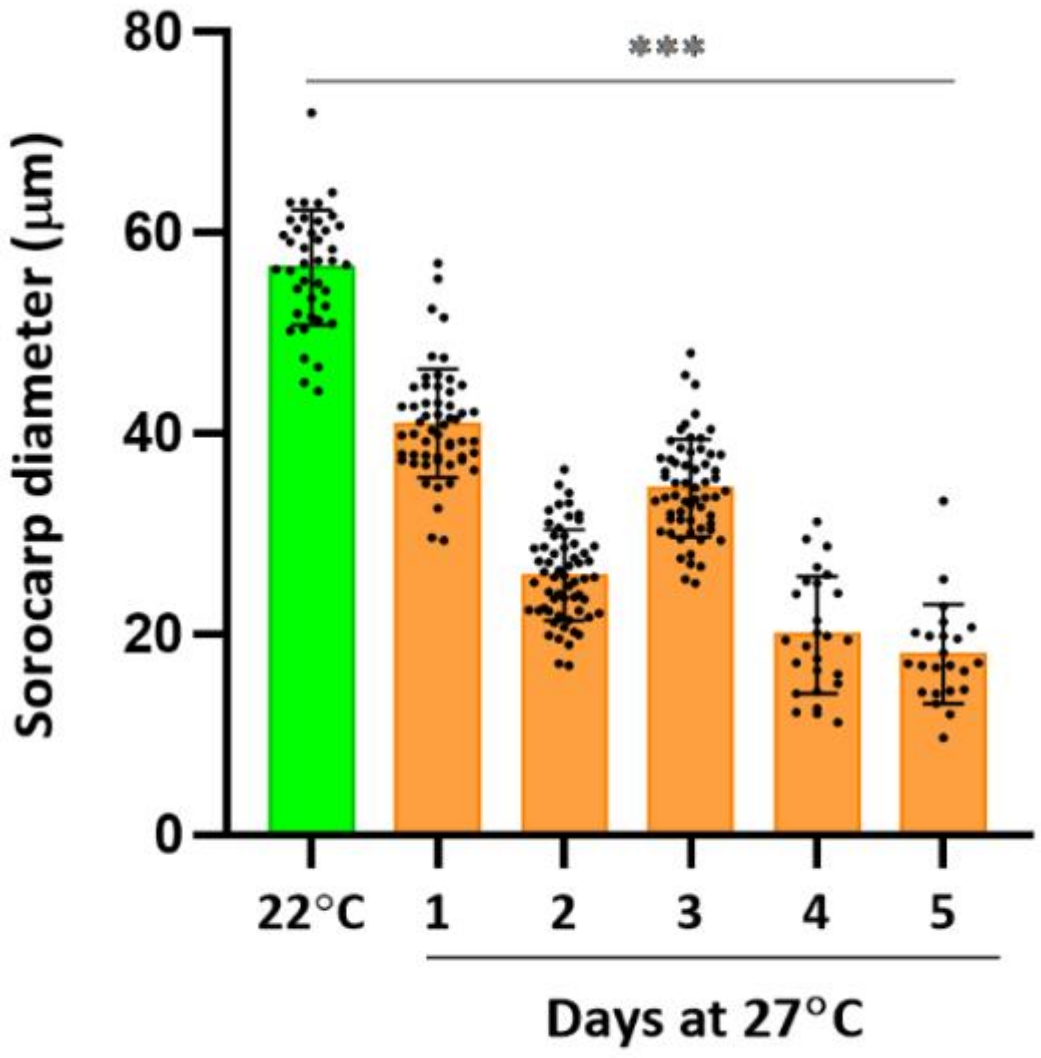

Supplemental Figure 2. Effect of heat stress duration on *D. discoideum* sorocarp size. The experiment was conducted as in Fig 4. Images were acquired with a ZEISS stereomicroscope at the end of experiment and used to measure sorocarp diameters. Data are presented as mean ± SD of 23 - 60 sorocarps per condition from two independent experiments. ***P < 0.001 relative to 22°C. ANOVA followed by Tukey's test.

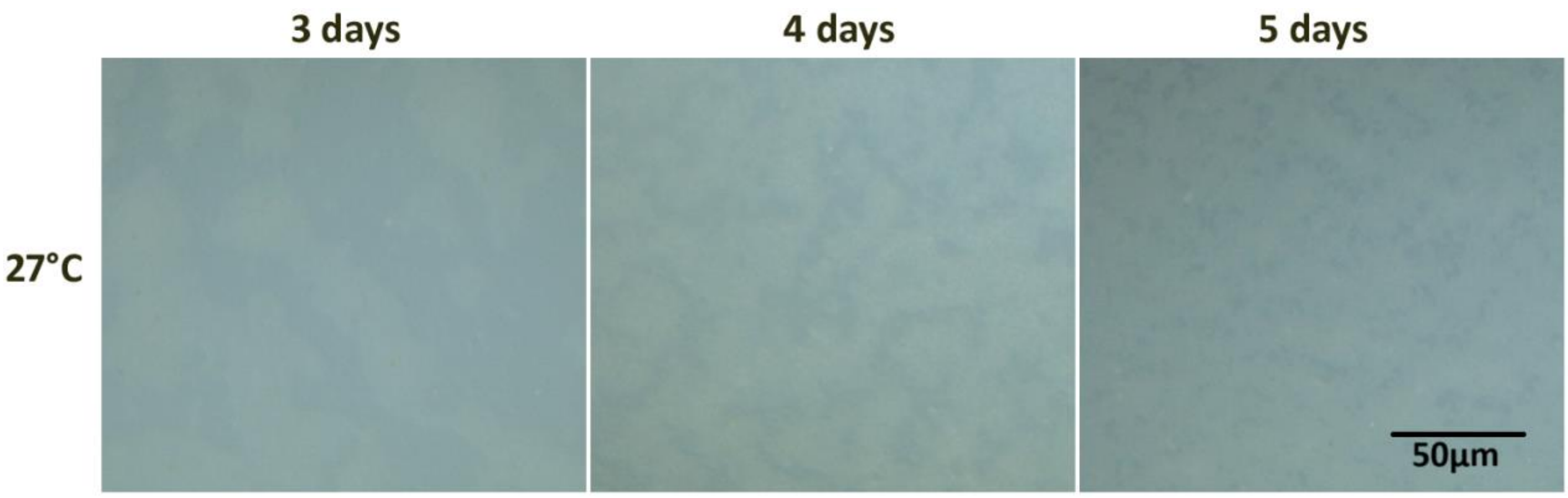

Supplemental Figure 3. Effect of heat stress duration on *D. discoideum* development. Vegetative cells were cultivated in suspension at 27°C, and samples were collected daily from 3 to 5 days. The cells were then allowed to develop on non-nutrient agar plates at 27°C. Images were captured after 30 hours using a ZEISS stereomicroscope.

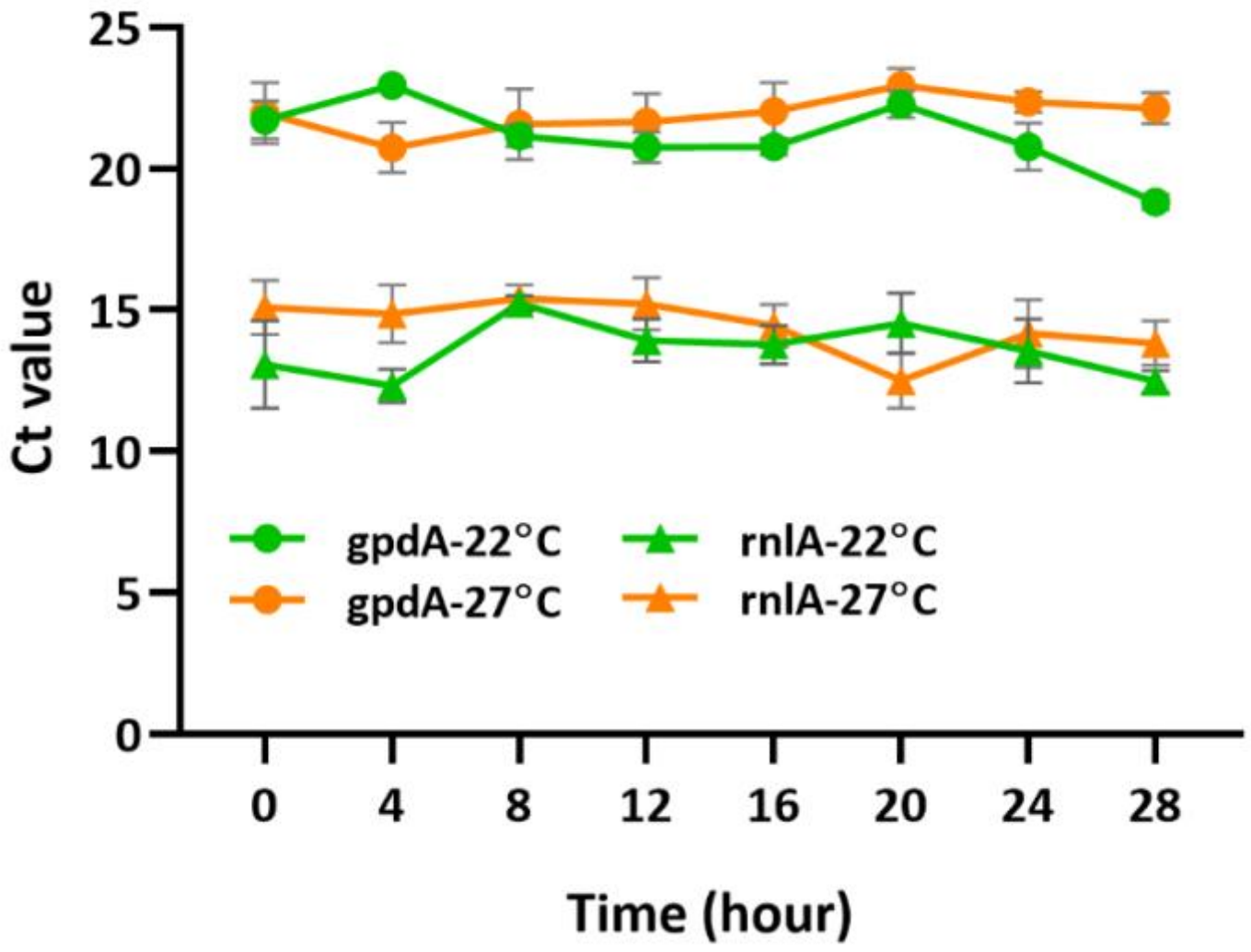

Supplemental Figure 4. Expression pattern of the reference genes *gpdA* and *rnlA* across the development cycle. Cells were grown at 22°C (green) or subjected to a simulated heatwave for 3 days at 27°C (orange) and allowed to develop on nitrocellulose filters at 22°C. Gene expression was determined by RT-qPCR. Data are mean ± SD of 6 (*gpdA*) or 4 (*rnlA*) determinations.

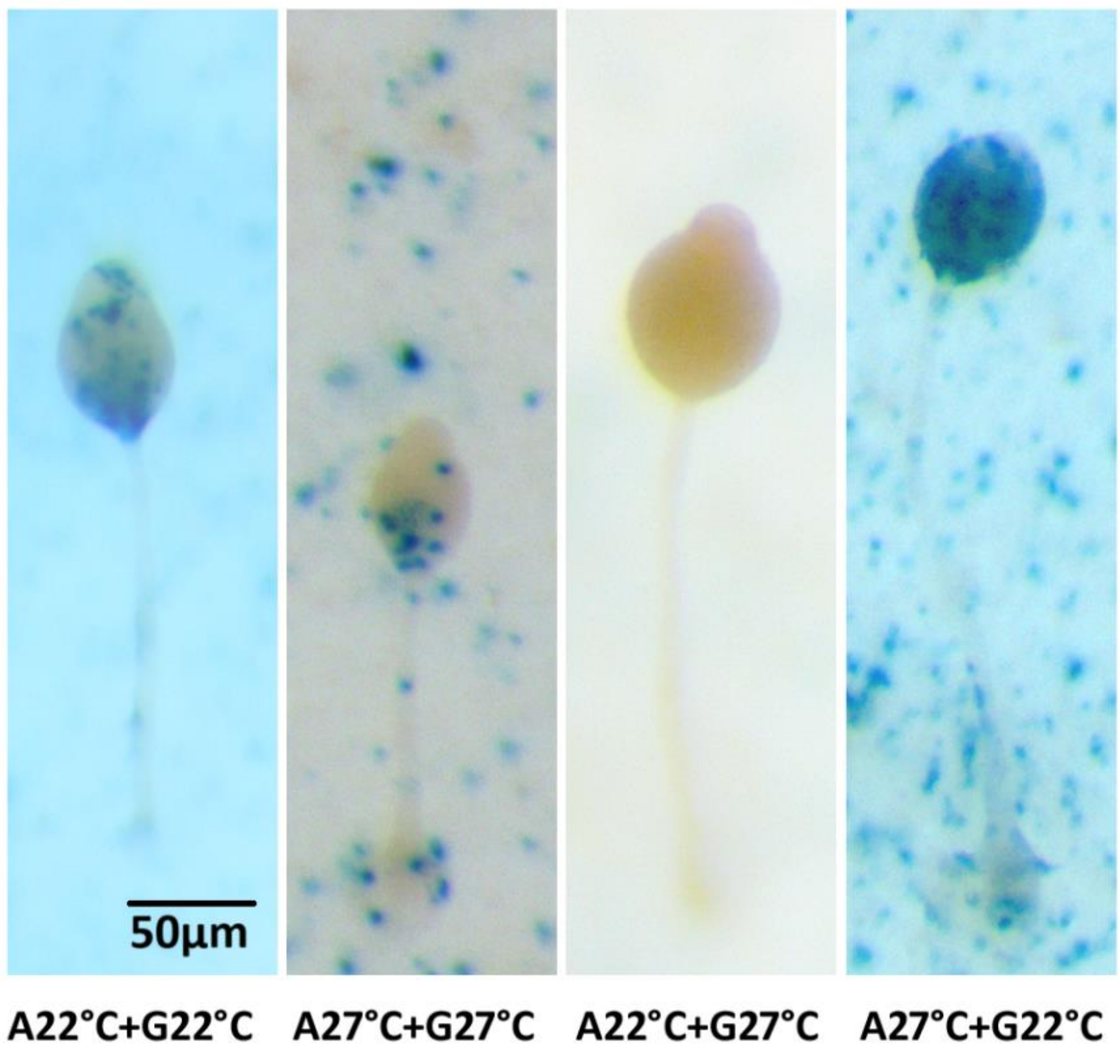

Supplemental Figure 5. Effects of a heatwave on cell fate during development using the A15-Gal reporter strain expressing the β-galactosidase gene under the control of the constitutive actin 15 promoter. AX2 (A) and A15-Gal (G) cells were grown at 22°C or 27°C for 3 days and then mixed in various combinations using a 5% proportion of reporter cells. The mixtures were allowed to develop synchronously on nitrocellulose filters at 22°C and processed as described in Figure 6A. Chimeric fruiting bodies are shown.

Supplemental Table 1. Statistical analysis of *D. discoideum* AX2 colony growth rate on agar with a *K. aerogenes* lawn at various temperatures (Figure 2C). Colony size was analyzed using a nonparametric generalized linear mixed model (GLMM) after determining that residuals were not normally distributed. A gamma GLMM with a log link function was applied. Random effects for replicate, plate number and colony number were included to control for nested dependencies within the experimental design. P values were calculated using a Tukey's multiple comparison test. ns, not significant; *P<0.05; **P<0.01; ***P < 0.001.

Table 1a. Random effects. Number of observations: 384. Groups: Replicate, 4; Plate number, 4; Colony number, 12. Dispersion estimate for gamma family (sigma^2): 0.263

Group	Name	Variance	Standard deviation
Replicate	Intercept	0.08693	0.29484
Plate Number	Intercept	0.00895	0.094619
Colony Number	Intercept	0.000038	0.006163

Table 1b. Conditional model after accounting for random factors replicate, plate number and colony number.

Predictor	Estimate	Standard error	Z value	Summary	P value
(Intercept)	-0.199	0.175	-1.136	n	0.256
Temperature 27°C	-0.386	0.0652	-5.92	***	3.22E-09
Temperature 30°C	-1.727	0.0648	-26.655	***	<2E-16
Days	0.29	0.0242	11.968	***	<2E-16

Table 1c. Post hoc test after accounting for random factors replicate, plate number and colony number.

Tukey's multiple comparisons test	Mean diff.	95.00% CI of diff.	Summary	Adjusted P value
D1:22°C vs. D1:27°C	0.1594	-0.2687 to 0.5875	ns	0.9868
D1:22°C vs. D1:30°C	0.5545	0.1367 to 0.9723	***	0.001
D1:22°C vs. D2:22°C	-0.7515	-1.169 to -0.3337	***	<0.001
D1:22°C vs. D2:27°C	-0.2939	-0.7221 to 0.1342	ns	0.5085
D1:22°C vs. D2:30°C	0.5394	0.1216 to 0.9572	**	0.0016
D1:22°C vs. D3:22°C	-1.636	-2.054 to -1.219	***	<0.001
D1:22°C vs. D3:27°C	-0.7206	-1.149 to -0.2925	***	<0.001
D1:22°C vs. D3:30°C	0.5303	0.1125 to 0.9481	**	0.0022
D1:22°C vs. D4:22°C	-2.555	-2.972 to -2.137	***	<0.001
D1:22°C vs. D4:27°C	-1.221	-1.649 to -0.7925	***	<0.001
D1:22°C vs. D4:30°C	0.5273	0.1095 to 0.9451	**	0.0024
D1:27°C vs. D1:30°C	0.3952	-0.03297 to 0.8233	ns	0.1025
D1:27°C vs. D2:22°C	-0.9109	-1.339 to -0.4828	***	<0.001
D1:27°C vs. D2:27°C	-0.4533	-0.8915 to -0.01514	*	0.0352
D1:27°C vs. D2:30°C	0.38	-0.04812 to 0.8081	ns	0.1384
D1:27°C vs. D3:22°C	-1.796	-2.224 to -1.368	***	<0.001
D1:27°C vs. D3:27°C	-0.88	-1.318 to -0.4418	***	<0.001
D1:27°C vs. D3:30°C	0.3709	-0.05721 to 0.7990	ns	0.1641

D1:27°C vs. D4:22°C	-2.714	-3.142 to -2.286	***	<0.001
D1:27°C vs. D4:27°C	-1.38	-1.818 to -0.9418	***	<0.001
D1:27°C vs. D4:30°C	0.3679	-0.06024 to 0.7960	ns	0.1733
D1:30°C vs. D2:22°C	-1.306	-1.724 to -0.8883	***	<0.001
D1:30°C vs. D2:27°C	-0.8485	-1.277 to -0.4204	***	<0.001
D1:30°C vs. D2:30°C	-0.01515	-0.4330 to 0.4026	ns	>0.9999
D1:30°C vs. D3:22°C	-2.191	-2.609 to -1.773	***	<0.001
D1:30°C vs. D3:27°C	-1.275	-1.703 to -0.8470	***	<0.001
D1:30°C vs. D3:30°C	-0.02424	-0.4420 to 0.3936	ns	>0.9999
D1:30°C vs. D4:22°C	-3.109	-3.527 to -2.691	***	<0.001
D1:30°C vs. D4:27°C	-1.775	-2.203 to -1.347	***	<0.001
D1:30°C vs. D4:30°C	-0.02727	-0.4451 to 0.3905	ns	>0.9999
D2:22°C vs. D2:27°C	0.4576	0.02946 to 0.8857	*	0.0245
D2:22°C vs. D2:30°C	1.291	0.8731 to 1.709	***	<0.001
D2:22°C vs. D3:22°C	-0.8848	-1.303 to -0.4670	***	<0.001
D2:22°C vs. D3:27°C	0.03091	-0.3972 to 0.4590	ns	>0.9999
D2:22°C vs. D3:30°C	1.282	0.8640 to 1.700	***	<0.001
D2:22°C vs. D4:22°C	-1.803	-2.221 to -1.385	***	<0.001
D2:22°C vs. D4:27°C	-0.4691	-0.8972 to -0.04097	*	0.0182
D2:22°C vs. D4:30°C	1.279	0.8610 to 1.697	***	<0.001
D2:27°C vs. D2:30°C	0.8333	0.4052 to 1.261	***	<0.001
D2:27°C vs. D3:22°C	-1.342	-1.771 to -0.9143	***	<0.001
D2:27°C vs. D3:27°C	-0.4267	-0.8649 to 0.01153	ns	0.0645
D2:27°C vs. D3:30°C	0.8242	0.3961 to 1.252	***	<0.001
D2:27°C vs. D4:22°C	-2.261	-2.689 to -1.832	***	<0.001
D2:27°C vs. D4:27°C	-0.9267	-1.365 to -0.4885	***	<0.001
D2:27°C vs. D4:30°C	0.8212	0.3931 to 1.249	***	<0.001
D2:30°C vs. D3:22°C	-2.176	-2.594 to -1.758	***	<0.001
D2:30°C vs. D3:27°C	-1.26	-1.688 to -0.8319	***	<0.001
D2:30°C vs. D3:30°C	-0.00909	-0.4269 to 0.4087	ns	>0.9999
D2:30°C vs. D4:22°C	-3.094	-3.512 to -2.676	***	<0.001
D2:30°C vs. D4:27°C	-1.76	-2.188 to -1.332	***	<0.001
D2:30°C vs. D4:30°C	-0.01212	-0.4299 to 0.4057	ns	>0.9999
D3:22°C vs. D3:27°C	0.9158	0.4876 to 1.344	***	<0.001
D3:22°C vs. D3:30°C	2.167	1.749 to 2.584	***	<0.001
D3:22°C vs. D4:22°C	-0.9182	-1.336 to -0.5004	***	<0.001
D3:22°C vs. D4:27°C	0.4158	-0.01236 to 0.8439	ns	0.0661
D3:22°C vs. D4:30°C	2.164	1.746 to 2.581	***	<0.001
D3:27°C vs. D3:30°C	1.251	0.8228 to 1.679	***	<0.001
D3:27°C vs. D4:22°C	-1.834	-2.262 to -1.406	***	<0.001
D3:27°C vs. D4:27°C	-0.5	-0.9382 to -0.06181	*	0.0108
D3:27°C vs. D4:30°C	1.248	0.8198 to 1.676	***	<0.001
D3:30°C vs. D4:22°C	-3.085	-3.503 to -2.667	***	<0.001
D3:30°C vs. D4:27°C	-1.751	-2.179 to -1.323	***	<0.001
D3:30°C vs. D4:30°C	-0.00303	-0.4208 to 0.4148	ns	>0.9999
D4:22°C vs. D4:27°C	1.334	0.9058 to 1.762	***	<0.001
D4:22°C vs. D4:30°C	3.082	2.664 to 3.500	***	<0.001
D4:27°C vs. D4:30°C	1.748	1.320 to 2.176	***	<0.001

Supplemental Table 2. Statistical analysis of *D. discoideum* AX2 fruiting body count on agar with a *K. aerogenes* lawn at various temperatures (Figure 3C). Fruiting body number was analyzed using a nonparametric GLMM after determining that residuals were not normally distributed. Random effects for replicate, plate number and colony number were included to control for nested dependencies within the experimental design. P values were calculated using a Tukey's multiple comparison test. ns, not significant; *P<0.05; **P<0.01; ***P < 0.001.

Table 2a. Random effects**.** Number of observations: 130. Groups: Replicate, 4; Plate number, 4; Colony number, 12. Dispersion parameter for nbinom2 family: 19.1

Groups	Name	Variance	Standard deviation
Replicate	(Intercept)	0.1118	0.3344
Plate number	(Intercept)	0.00507	0.07121
Colony number	(Intercept)	1.59E-10	1.26E-05

Table 2b. Conditional model after accounting for random factors replicate, plate number and colony number.

	Estimate	Standard error	z value	Summary	Pr(>|z|)
(Intercept)	3.46028	0.18035	19.186	***	< 2E-16
27°C	-0.23015	0.07948	-2.896	**	0.00378
30°C	-3.25419	0.16848	-19.315	***	< 2E-16
30°C Recovery	-0.74507	0.08357	-8.915	***	< 2E-16

Table 2c. Post hoc test after accounting for random factors replicate, plate number and colony number.

Tukey's multiple comparisons test	Estimate	Standard error	z ratio	Summary	P value
22°C vs. 27°C	0.23	0.0795	2.896	*	0.0198
22°C vs. 30°C	3.254	0.168	19.315	***	<0.0001
22°C vs. 30°C Recovery	0.745	0.0836	8.915	***	<0.0001
27°C vs. 30°C	3.024	0.17	17.802	***	<0.0001
27°C vs. 30°C Recovery	0.515	0.0849	6.062	***	<0.0001
30°C vs. 30°C Recovery	-2.509	0.171	-14.633	***	<0.0001

Supplemental Table 3. Statistical analysis of *D. discoideum* AX2 spore yield on non-nutrient agar after growth at 27°C for various periods of time (Figure 4C). P values were calculated using mixed-effects ANOVA followed by Tukey's multiple comparison test. ns, not significant; *P<0.05; **P<0.01; ***P < 0.001.

Tukey's multiple comparisons test	Mean diff.	95.00% CI of diff.	Summary	Adjusted P value
22°C vs. 1	0.1131	-0.1012 to 0.3275	ns	0.5868
22°C vs. 2	0.2492	0.03488 to 0.4636	*	0.0162
22°C vs. 3	0.4083	0.1939 to 0.6226	***	<0.001
22°C vs. 4	0.5689	0.3546 to 0.7833	***	<0.001
22°C vs. 5	0.6958	0.4814 to 0.9101	***	<0.001
1 vs. 2	0.1361	-0.07824 to 0.3505	ns	0.3909
1 vs. 3	0.2952	0.08081 to 0.5095	**	0.0033
1 vs. 4	0.4558	0.2414 to 0.6701	***	<0.001
1 vs. 5	0.5826	0.3683 to 0.7970	***	<0.001
2 vs. 3	0.1591	-0.05530 to 0.3734	ns	0.2349
2 vs. 4	0.3197	0.1053 to 0.5340	**	0.0014
2 vs. 5	0.4465	0.2322 to 0.6609	***	<0.001
3 vs. 4	0.1606	-0.05372 to 0.3750	ns	0.2261
3 vs. 5	0.2875	0.07313 to 0.5018	**	0.0043
4 vs. 5	0.1268	-0.08750 to 0.3412	ns	0.4667

Supplemental Table 4. Statistical analysis of the effects of a heatwave on expression of developmental marker genes. A beta regression approach was used to accommodate the bounded nature of the data due to min-max normalisation. A GLMM was fitted using the glmmTMB package in R, with gene type (early development, prestalk, prespore), temperature (22°C, 27°C) and their interaction as fixed effects. To account for variation across genes and experimental replicates, random intercepts for gene and replicate were included. Post hoc comparisons were performed using estimated marginal means (emmeans), and results were visualized using mean response plots with 95% confidence intervals. ns, not significant; *P<0.05; **P<0.01; ***P < 0.001.

Table 4a. Linear model analysis of reference gene expression based on plate number. Because samples were run in separate multiwell plates, each including one of the reference genes, either *gpdA* or *rnlA*, it was first verified that the plate number was not a significant predictor of reference gene expression level.

Reference gene	Parameter	Estimate	Standard error	t value	Summary	P value
gpdA	(Intercept)	20.9543	0.4866	43.062	**	< 2E-16
gpdA	Plate	0.1021	0.2253	0.453	ns	0.653
rnlA	(Intercept)	16.6353	1.9095	8.712	**	1.02E-09
rnlA	Plate	-0.6711	0.4217	-1.591	ns	0.122

Table 4b. Conditional model. 22°C was used as the reference temperature and early development as the reference for development stage.

Parameter	Estimate	Standard error	Z value	Summary	Pr(>|z|)
(Intercept)	-0.1322	0.1391	-0.95	ns	0.34199
Prespore	-0.7299	0.2679	-2.725	**	0.00644
Prestalk	-0.3306	0.3621	-0.913	ns	0.36128
27°C	-0.3832	0.1953	-1.962	*	0.04971
Prespore: 27°C	-0.105	0.3665	-0.286	ns	0.77457
Prestalk: 27°C	-0.4207	0.4918	-0.856	ns	0.39223

Table 4c. Post hoc comparisons. The data was analyzed to investigate the effects of temperature (22°C vs. 27°C) on expression levels of genes known to be active at different developmental stages using estimated marginal means.

Gene Type	Odds ratio	Standard error	df	Z ratio	Summary	P value
Early development	1.47	0.286	Inf	1.962	*	0.0497
Prespore	1.63	0.507	Inf	1.57	ns	0.1165
Prestalk	2.23	1.01	Inf	1.776	ns	0.0757

Supplemental Table 5. Statistical analysis of *D. discoideum* group fitness of AX2 (A) and A6-Gal (G) grown at 22°C or 27°C and developed at 22°C either clonally or in chimeric mixes (Figure 7A). P values were calculated using mixed-effects ANOVA followed by Tukey's multiple comparison test. ns, not significant; *P<0.05; **P<0.01; ***P < 0.001.

Tukey's multiple comparisons test	Mean diff.	95.00% CI of diff.	Summary	Adjusted P value
A22°C vs. A27°C	0.6117	0.4376 to 0.7857	***	<0.001
A22°C vs. G22°C	0.4181	0.2440 to 0.5921	***	<0.001
A22°C vs. G27°C	0.7228	0.5487 to 0.8969	***	<0.001
A22°C vs. A22°C+G22°C	0.09611	-0.07797 to 0.2702	ns	0.6681
A22°C vs. A22°C+G27°C	0.3953	0.2212 to 0.5694	***	<0.001
A22°C vs. A27°C+G22°C	0.3597	0.1856 to 0.5338	***	<0.001
A22°C vs. A27°C+G27°C	0.6117	0.4376 to 0.7857	***	<0.001
A27°C vs. G22°C	-0.1936	-0.3677 to -0.01953	*	0.019
A27°C vs. G27°C	0.1111	-0.06297 to 0.2852	ns	0.49
A27°C vs. A22°C+G22°C	-0.5156	-0.6896 to -0.3415	***	<0.001
A27°C vs. A22°C+G27°C	-0.2164	-0.3905 to -0.04231	**	0.0055
A27°C vs. A27°C+G22°C	-0.2519	-0.4260 to -0.07786	***	0.0007
A27°C vs. A27°C+G27°C	0	-0.1741 to 0.1741	ns	>0.9999
G22°C vs. G27°C	0.3047	0.1306 to 0.4788	***	<0.001
G22°C vs. A22°C+G22°C	-0.3219	-0.4960 to -0.1479	***	<0.001
G22°C vs. A22°C+G27°C	-0.02278	-0.1969 to 0.1513	ns	>0.9999
G22°C vs. A27°C+G22°C	-0.05833	-0.2324 to 0.1157	ns	0.9645
G22°C vs. A27°C+G27°C	0.1936	0.01953 to 0.3677	*	0.019
G27°C vs. A22°C+G22°C	-0.6267	-0.8007 to -0.4526	***	<0.001
G27°C vs. A22°C+G27°C	-0.3275	-0.5016 to -0.1534	***	<0.001
G27°C vs. A27°C+G22°C	-0.3631	-0.5371 to -0.1890	***	<0.001
G27°C vs. A27°C+G27°C	-0.1111	-0.2852 to 0.06297	ns	0.49
A22°C+G22°C vs. A22°C+G27°C	0.2992	0.1251 to 0.4732	***	<0.001
A22°C+G22°C vs. A27°C+G22°C	0.2636	0.08953 to 0.4377	***	0.0003
A22°C+G22°C vs. A27°C+G27°C	0.5156	0.3415 to 0.6896	***	<0.001
A22°C+G27°C vs. A27°C+G22°C	-0.03556	-0.2096 to 0.1385	ns	0.9981
A22°C+G27°C vs. A27°C+G27°C	0.2164	0.04231 to 0.3905	**	0.0055
A27°C+G22°C vs. A27°C+G27°C	0.2519	0.07786 to 0.4260	***	0.0007

Supplemental Table 6. Statistical analysis of *D. discoideum* strain fitness in chimera mixes. Strain fitness was calculated for the AX2 (A) non-labeled strain and the A6-Gal (G) labeled strain (Figure 7B). P values were calculated using mixed-effects ANOVA followed by Sidak's multiple comparison test. ns, not significant; **P<0.01; ***P < 0.001.

Sidak's multiple comparisons test	Mean diff.	95.00% CI of diff.	Summary	Adjusted P value
A22°C+G22°C:Fitness of labelled strain vs. A22°C+G22°C:Fitness of non labelled strain	-1.027	-1.259 to -0.7952	***	<0.001
A22°C+G22°C:Fitness of labelled strain vs. A27°C+G27°C:Fitness of labelled strain	0.2804	0.04828 to 0.5126	**	0.0059
A22°C+G22°C:Fitness of labelled strain vs. A27°C+G27°C:Fitness of non labelled strain	-0.2778	-0.5099 to -0.04562	**	0.0067
A22°C+G22°C:Fitness of labelled strain vs. A22°C+G27°C:Fitness of labelled strain	0.3196	0.08740 to 0.5517	***	0.0009
A22°C+G22°C:Fitness of labelled strain vs. A22°C+G27°C:Fitness of non labelled strain	-0.7486	-0.9807 to -0.5164	***	<0.001
A22°C+G22°C:Fitness of labelled strain vs. A27°C+G22°C:Fitness of labelled strain	-0.809	-1.041 to -0.5768	***	<0.001
A22°C+G22°C:Fitness of labelled strain vs. A27°C+G22°C:Fitness of non labelled strain	0.3083	0.07617 to 0.5405	**	0.0016
A22°C+G22°C:Fitness of non labelled strain vs. A27°C+G27°C:Fitness of labelled strain	1.308	1.076 to 1.540	***	<0.001
A22°C+G22°C:Fitness of non labelled strain vs. A27°C+G27°C:Fitness of non labelled strain	0.7496	0.5174 to 0.9817	***	<0.001
A22°C+G22°C:Fitness of non labelled strain vs. A22°C+G27°C:Fitness of labelled strain	1.347	1.115 to 1.579	***	<0.001
A22°C+G22°C:Fitness of non labelled strain vs. A22°C+G27°C:Fitness of non labelled strain	0.2788	0.04662 to 0.5109	**	0.0064
A22°C+G22°C:Fitness of non labelled strain vs. A27°C+G22°C:Fitness of labelled strain	0.2183	-0.01383 to 0.4505	ns	0.0871
A22°C+G22°C:Fitness of non labelled strain vs. A27°C+G22°C:Fitness of non labelled strain	1.336	1.104 to 1.568	***	<0.001
A27°C+G27°C:Fitness of labelled strain vs. A27°C+G27°C:Fitness of non labelled strain	-0.5582	-0.7904 to -0.3261	***	<0.001
A27°C+G27°C:Fitness of labelled strain vs. A22°C+G27°C:Fitness of labelled strain	0.03911	-0.1930 to 0.2713	ns	>0.9999
A27°C+G27°C:Fitness of labelled strain vs. A22°C+G27°C:Fitness of non labelled strain	-1.029	-1.261 to -0.7968	***	<0.001

A27°C+G27°C:Fitness of labelled strain vs. A27°C+G22°C:Fitness of labelled strain	-1.089	-1.322 to -0.8573	***	<0.001
A27°C+G27°C:Fitness of labelled strain vs. A27°C+G22°C:Fitness of non labelled strain	0.02789	-0.2043 to 0.2600	ns	>0.9999
A27°C+G27°C:Fitness of non labelled strain vs. A22°C+G27°C:Fitness of labelled strain	0.5973	0.3652 to 0.8295	***	<0.001
A27°C+G27°C:Fitness of non labelled strain vs. A22°C+G27°C:Fitness of non labelled strain	-0.4708	-0.7029 to -0.2386	***	<0.001
A27°C+G27°C:Fitness of non labelled strain vs. A27°C+G22°C:Fitness of labelled strain	-0.5312	-0.7634 to -0.2991	***	<0.001
A27°C+G27°C:Fitness of non labelled strain vs. A27°C+G22°C:Fitness of non labelled strain	0.5861	0.3540 to 0.8183	***	<0.001
A22°C+G27°C:Fitness of labelled strain vs. A22°C+G27°C:Fitness of non labelled strain	-1.068	-1.300 to -0.8360	***	<0.001
A22°C+G27°C:Fitness of labelled strain vs. A27°C+G22°C:Fitness of labelled strain	-1.129	-1.361 to -0.8964	***	<0.001
A22°C+G27°C:Fitness of labelled strain vs. A27°C+G22°C:Fitness of non labelled strain	-0.01122	-0.2434 to 0.2209	ns	>0.9999
A22°C+G27°C:Fitness of non labelled strain vs. A27°C+G22°C:Fitness of labelled strain	-0.06044	-0.2926 to 0.1717	ns	>0.9999
A22°C+G27°C:Fitness of non labelled strain vs. A27°C+G22°C:Fitness of non labelled strain	1.057	0.8247 to 1.289	***	<0.001
A27°C+G22°C:Fitness of labelled strain vs. A27°C+G22°C:Fitness of non labelled strain	1.117	0.8852 to 1.349	***	<0.001

Supplemental Table 7. Statistical analysis of *D. discoideum* relative within-group fitness of the A6-Gal (G) strain in chimeric mixes with AX2 (A) (Figure 7C). P values were calculated using mixed-effects ANOVA followed by Tukey's multiple comparison test. ns, not significant; $^{**}P<0.01$; $^{***}P < 0.001$.

Tukey's multiple comparisons test	Mean diff.	95.00% CI of diff.	Summary	Adjusted P value
A22°C+G22°C vs. A27°C+G27°C	0.1121	-0.03578 to 0.2599	ns	0.1902
A22°C+G22°C vs. A22°C+G27°C	0.3194	0.1716 to 0.4673	***	<0.001
A22°C+G22°C vs. A27°C+G22°C	-1.456	-1.604 to -1.308	***	<0.001
A27°C+G27°C vs. A22°C+G27°C	0.2074	0.05951 to 0.3552	**	0.0033
A27°C+G27°C vs. A27°C+G22°C	-1.568	-1.716 to -1.420	***	<0.001
A22°C+G27°C vs. A27°C+G22°C	-1.775	-1.923 to -1.628	***	<0.001
